# Tumor-penetrating nanoplatform with ultrasound “unlocking” for cascade synergistic therapy and visual feedback under hypoxia

**DOI:** 10.1186/s12951-023-01765-x

**Published:** 2023-01-25

**Authors:** Zhuoyan Xie, Junrui Wang, Yuanli Luo, Bin Qiao, Weixi Jiang, Leilei Zhu, Haitao Ran, Zhigang Wang, Wei Zhu, Jianli Ren, Zhiyi Zhou

**Affiliations:** 1 Department of Ultrasound, Chongqing General Hospital, Chongqing, 401147 China; 2 Chongqing Key Laboratory of Ultrasound Molecular Imaging, The Second Affiliated Hospital of Chongqing Medical University, Chongqing, 400010 China; 3 Department of Radiology, The Second Affiliated Hospital of Chongqing Medical University, Chongqing, 400010 China; 4 Depatment of General Practice, Chongqing General Hospital, Chongqing, 401147 China; 5 Depatment of Medical College, Hubei University for Nationalities, Enshi, 445000 Hubei China

**Keywords:** Nanoultrasonic biomedicine, Acoustic droplet vaporization, Nanoplatform-based cascade engineering, O_2_ supply, Tumor-penetrating peptide

## Abstract

**Background:**

Combined therapy based on the effects of cascade reactions of nanoplatforms to combat specific solid tumor microenvironments is considered a cancer treatment strategy with transformative clinical value. Unfortunately, an insufficient O_2_ supply and the lack of a visual indication hinder further applications of most nanoplatforms for solid tumor therapy.

**Results:**

A visualizable nanoplatform of liposome nanoparticles loaded with GOD, H(Gd), and PFP and grafted with the peptide tLyP-1, named _tLyP-1_H(Gd)-GOD@PFP, was constructed. The double-domain peptide tLyP-1 was used to specifically target and penetrate the tumor cells; then, US imaging, starvation therapy and sonodynamic therapy (SDT) were then achieved by the ultrasound (US)-activated cavitation effect under the guidance of MR/PA imaging. GOD not only deprived the glucose for starvation therapy but also produced H_2_O_2_, which in coordination with ^1^O_2_ produced by H(Gd), enable the effects of SDT to achieve a synergistic therapeutic effect. Moreover, the synergistic therapy was enhanced by O_2_ from PFP and low-intensity focused ultrasound (LIFU)-accelerated redox effects of the GOD. The present study demonstrated that the nanoplatform could generate a 3.3-fold increase in ROS, produce a 1.5-fold increase in the maximum rate of redox reactions and a 2.3-fold increase in the O_2_ supply in vitro*,* and achieve significant tumor inhibition in vivo.

**Conclusion:**

We present a visualizable nanoplatform with tumor-penetrating ability that can be unlocked by US to overcome the current treatment problems by improving the controllability of the O_2_ supply, which ultimately synergistically enhanced cascade therapy.

**Supplementary Information:**

The online version contains supplementary material available at 10.1186/s12951-023-01765-x.

## Introduction

A sensitive redox state and high level of glucose consumption are unique features of the metabolic microenvironment of solid tumors [1–4]. These characteristics suggest that the use of the noninvasive therapeutic potential of reactive oxygen species (ROS)-producing and glucose-consuming nanoplatforms provides synergistic cascade treatment strategies for solid tumors; this subject is an important topic in current biomedical research. Starvation therapy emphasizes the use of exogenous nutrient blockers to cut off the energy supply of cancer cells to cause their death by starvation; for example, glucose oxidase (GOD) is a natural endogenous oxidoreductase, that reduces glucose to gluconic acid and hydrogen peroxide (H_2_O_2_) using O_2_ and H_2_O as oxidants. The GOD reaction not only cuts off an energy source of the tumor cells to achieve their purpose of starvation but also changes the redox balance of malignant tumor cells [5, 6]. Catalytic therapy refers to highly selective and efficient catalytic production of toxic ROS, such as H_2_O_2_, singlet oxygen (^1^O_2_), and hydroxyl radicals (•OH), through O_2_-dependent catalytic reactions to change the redox balance in a pathological tissue [7]. These approaches include sonocatalytic therapy (sonodynamic therapy, SDT), which utilizes a sonosensitizer (such as hematoporphyrin) that undergoes energy level transition upon stimulation with ultrasonic (US) energy to stimulate a change in valence and ultimately release an excess of ROS, especially ^1^O_2_ [8, 9]. A synergistic treatment strategy combining these two factors can compensate for the intrinsic defects of the corresponding treatments or synergistically enhance the efficacy of these treatments. However, the inherent hypoxic microenvironment of malignant tumors is the natural obstacle for both approaches and seriously hinders further development of nanoplatform-based cascade engineering. [10, 11]

Considerable effort has been devoted to optimizing nanoplatforms by overcoming tumor hypoxia using nanoplatforms with O_2_ carriers (Hb and non-Hb) and generators (catalase, water splitting, and metals) by nanozymes [12–15]. However, the treatments remain restricted by partial oxygen leakage before these platforms can reach hypoxic areas [16, 17]. On the one hand, sequential biological barriers result in limited tumor penetration and low endocytosis of a nanoplatform. On the other hand, these types of studies rely on the passive natural behavior of the nanoplatform that lacks artificial guidance and visual monitoring. In brief, the design of an optimized nanoplatform to achieve the full delivery of O_2_ and enhanced relevant therapeutic effects should include (i) nanoplatforms for excellent tumor penetration depth and endocytosis, (ii) a controlled O_2_-supplying nanoplatform-vehicle and visually monitored system, and (iii) a nanoplatform-based cascade therapeutic strategy, providing a significant therapeutic effect on hypoxic tumors.

Nanoultrasonic biomedicines, including low-diffusion liquid perfluorocarbon (PFC)-based nanodroplets, have been designed for US-activated specific drug release, molecular imaging, SDT, etc [18–22]. Moreover, after US activation, the nanodroplets reach a certain threshold; thus, a large amount of liquid PFC is converted into a gaseous state, and a final rupture occurs due to vibration and expansion, resulting in an ultrasonic cavitation effect, which is also known as acoustic droplet vaporization (ADV), to achieve highly selective drug release and responsive imaging in a pathological tissue [23, 24]. Additionally, various formulations of liquid PFC, which have been reported to carry more than 1.5 times more O_2_ than blood (25 °C, 1 atm), have been extensively explored as artificial blood substitutes [25, 26]. Considering the weak physical van der Waals interactions between O_2_ and PFC molecules, US has been the primary approach used to trigger O_2_ release. Therefore, the use of nanoultrasonic medicine enables the establishment of a nanoplatform unlocked by US, and it is not only possible to integrate controllable O_2_ supply with a visual imaging system but also to achieve specific hypoxic tumor synergistic cascade therapy.

**Scheme 1:**
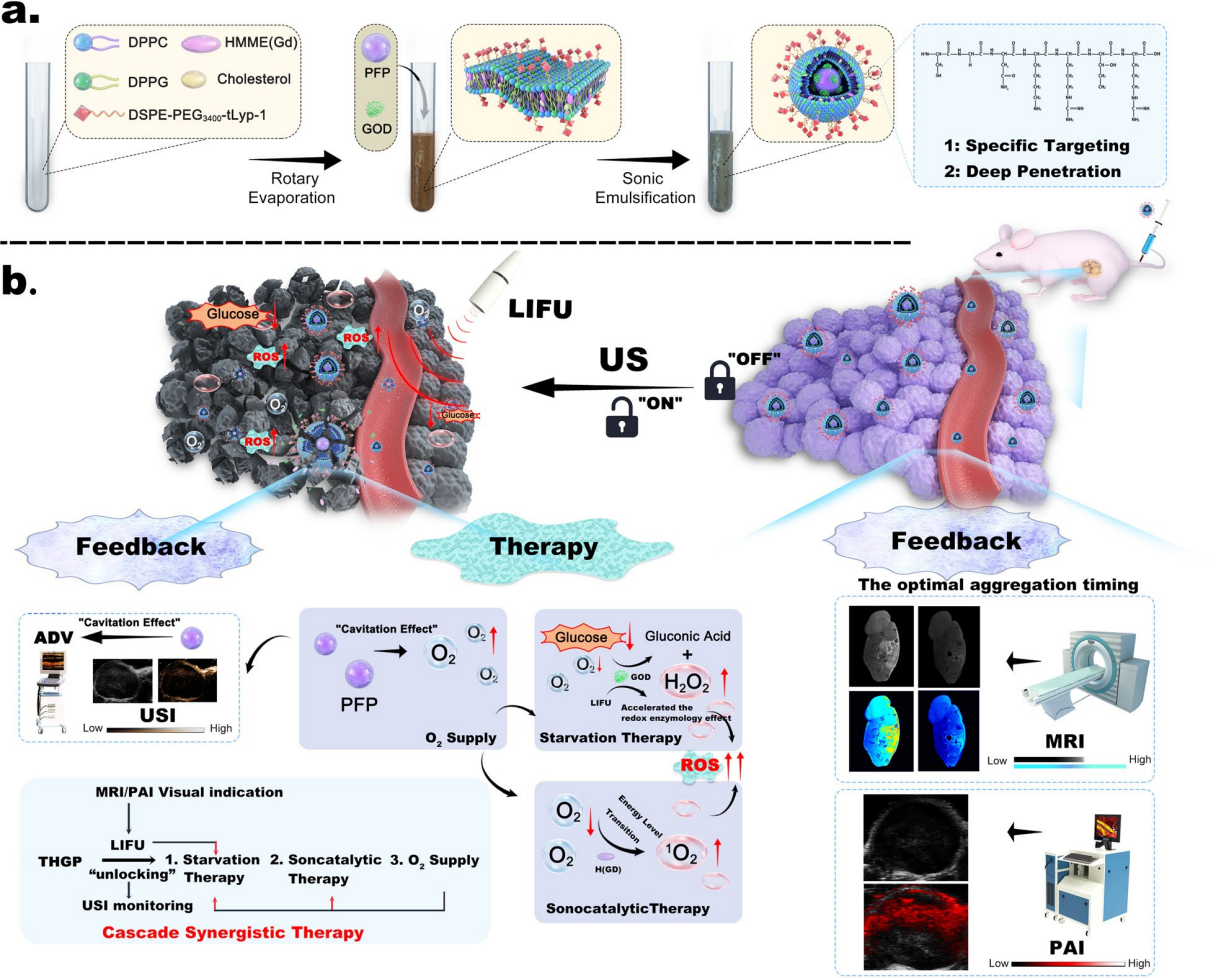
**a** Construction of the tumorspecific visualization nanoplatform (THGP): liposomes are loaded with a fluorocarbon (PFP), gadolinium-labeled hematoporphyrin monomethyl ether (H(Gd)) and glucose oxidase (GOD), and the tLyP-1 peptide is grafted onto the liposome shell. **b** Capabilities of the nanoplatform for synergistic cascade therapy: deep penetration of the tumor, PA/MR/US imaging, O_2_ supply, starvation therapy and SDT

We constructed a tumor-specific visualization nanoplatform (_tLyP-1_H(Gd)-GOD@PFP, THGP) by introducing nanoultrasonic biomedicine. As shown in Scheme. 1a, liposome-based nanoplatforms can encapsulate hydrophobic drugs (gadolinium-labeled hematoporphyrin monomethyl ether (H(Gd))) within their bimolecular hydrophobic layer and encapsulate hydrophilic drugs (GOD and perfluoropentane (PFP)) in the hydrophilic core. Nanoplatfrom-richened liposomes have the characteristic structure of a bimolecular phospholipid layer similar to the cell membrane, and can strengthen the lipid bilayer membrane by modifying cholesterol, and reducing membrane flow, and reducing the leakage rate, which significantly reduce uncontrolled starvation therapy and catalytic reactions [27, 28]. The tLyP-1 peptide was grafted onto the liposome shell, which contains two domains (tumor-homing domain and Cend-R domain); the tumor-homing domain specifically binds to the neuropilin 1 (NRP-1) target on a tumor, and the Cend-R domain can promote internalization by the cells and tissue penetration of the nanoplatforms [29, 30]. Importantly, the strategy of US-unlocked cascade therapy is realized through irradiation by low-intensity focused ultrasound (LIFU); this approach not only controls the O_2_ supply and visualization of a lesion site but also enhances the efficiency of synergistic treatment of hypoxic tumors (Scheme. 1b). In summary, we validated this process using in vitro*/*in vivo tumor models of human breast cancer cells (MDA-MB-231), which are characterized by a few specific targets and have high relevance to clinical applications of US imaging. The present study plays a positive role in promoting the cross-integration and clinical transformation of nanoplatform-based cascade engineering and ultrasonic medicines.

## Methods

### Materials

H(Gd) was obtained from Medixin Co. (Beijing, China). GOD (100 units/mg protein) and cholesterol (CH) were purchased from Qiangyao Co. (Shanghai, China). Dipalmitoyl phosphatidylcholine (DPPC), dipalmitoyl phosphatidylglycerol (DPPG), and ethanolamine-polyethylene glycol (DSPE-PEG_3400_) were purchased from Ruixi Co. (Xi’an, China). DSPE-PEG_3400_-tLyP-1 was synthesized via a maleimide-thiol coupling reaction by Protein Way Co. (Chongqing, China). PFP was purchased from ElfAtochem (France).

### Synthesis of the _tLyP-1_H(Gd)-GOD@PFP

The liposomes were prepared by a previously reported double emulsification method. [31] More detailed parameters are described in the Additional file 1.

### Physical and chemical properties of _tLyP-1_H(Gd)-GOD@PFP

The morphology of THGP was studied by transmission electron microscopy (TEM, JEM 1200EX, Jeol, Japan). The particle size distribution and zeta potential of the samples were measured using a laser particle size analyzer (ZS90; Malvern Instruments, Ltd., USA). A Fourier transform infrared spectrometer (FTIR; iS10; Thermo Nicolet, USA) was used to assess the changes in chemical bonding. Nuclear magnetic resonance (NMR; Bruker AVANCE III 600; Bruker, Germany) was used to evaluate F in THGP. The encapsulation efficiency of H(Gd) in THGP was determined by measuring the unbound concentration of H(Gd) in the supernatant by UV–VIS-NIR spectroscopy (UV-3600; Shimazu, Japan). The SDS–polyacrylamide gel (SDS‒PAGE) was used to verify the efficiency of GOD loading. In addition, THGP was dissolved and demulsified in DMSO, and the amount of GOD was measured by high-performance liquid chromatography (HPLC; Agilent 1200; Agilent Technologies, USA). The details of the drug encapsulation efficiency (EE) evaluation and loading capacity (LC) are described in Additional file 1: Formulations 1 and 2 (SI).

### Ultrasound unlocking of the cascade reactions

#### Redox reaction of glucose

To explore the effect of LIFU irradiation on the redox reaction, a fixed concentration of THGP and a fixed amount of glucose solution were irradiated by LIFU (pulse mode; 1.6 W/cm^2^; 60–300 s); sodium acetate (NaAc) buffer solution (3 mL; 20 mM; pH = 5.2) containing 3,3',5,5'-tetramethylbenzidine (TMB) (3.2 mM) and glucose solutions of variable concentrations were added at the same time. Then, the changes in pH induced by GOD were measured over time by a microprocessor pH meter (Mettler Toledo le438; Mettler Toledo, Switzerland Confederation). According to the same treatment protocol, the ability of each group to produce H_2_O_2_ was detected by an H_2_O_2_ assay kit (Solarbio Science & Technology, Beijing, China).

#### Catalytic reaction

The production of ^1^O_2_ by THGP was measured using a ^1^O_2_-sensor green (SOSG) probe (Invitrogen, USA). The output of ^1^O_2_ by THGP was measured by electron spin resonance spectroscopy (ESR; Bruker A300-10/12; Bruker, Germany).

#### O_2_ supply

The production of O_2_ by THGP was measured by a dissolved oxygen meter (B949712069, Mettler-Toledo Instruments Co., Ltd.). Briefly, after THGP was treated with LIFU (pulse mode; 1.6 W/cm^2^, 0–5 min), the concentration of O_2_ in the solution was continuously measured, and a curve was constructed. The THGP + LIFU groups with gradient concentrations (0.25–1 mg mL^−1^) were treated in a similar manner, and the O_2_ concentration was determined. Detailed parameters of the protocols are described in the Additional file 1: Experimental (SI).

### Cell experiment

#### Cell culture

Human umbilical vein endothelial cells (HUVECs) and MDA-MB-231 cells lines were obtained from Chongqing Key Laboratory of Ultrasound Molecular Imaging (Chongqing, China). The details are described in the Additional file 1: Experimental (SI).

#### Cellular internalization and permeability

MDA-MB-231 cells and HUVECs were seeded at a density of 1*10^5^ cells/well in a confocal dish for 24 h. Then, DiI-labeled THGP or HGP was added to the dishes for various time intervals (0.5, 1, 2, and 4 h). To explore the penetration ability, MDA-MB-231 cells were seeded at a density of 1*10^5^ cells/well in spheroid microplates (Corning, USA) to obtain a three-dimensional cell sphere model. After incubation for 7 d, the spheroids had formed, and DiI-labeled THGP or HGP in Hoechst 33,342 solution was added to the wells for 2 h. Then, fluorescence imaging of the 3D spheroids was performed by confocal laser scanning microscopy (CLSM; A1R-si; Nikon, Japan).

#### Intracellular ROS generation and alleviation of hypoxia

A 2’,7’-dichlorofluorescin diacetate (DCFH-DA; Beyotime, Shanghai, China) assay was used to measure ROS production inside MDA-MB-231 cells after various treatments. The specific treatment groups were as follows: PBS, LIFU, THGP, TGP + LIFU, THP + LIFU and THGP + LIFU. Irradiation with LIFU was carried out in the corresponding groups (pulse mode; 1.6 W/cm^2^; 4 min). MDA-MB-231 cells were seeded in T25 cell culture flasks at a density of 5*10^6^ cells/flask for 24 h under hypoxic conditions. The cells were then subjected to various treatments, and cellular proteins were extracted for Western blot (WB) analysis.

#### Evaluation of the cytotoxicity of synergistic therapy

To evaluate the effects of hypoxia and normoxia on cytotoxicity, each treatment group was placed in environments with different O_2_ concentrations, and the cell viability was detected by CCK-8 assay. The experimental details are described in the Additional file 1: Experimental (SI).

### Biosafety evaluation

#### Tumor-bearing nude mice

All animal procedures were carried out according to the Guidelines of the Institutional Animal Care and Use Committee of Chongqing Medical University and approved by the Animal Ethics Committee of Chongqing Medical University. Female nude mice (6 ~ 8 weeks, 17 ~ 20 g) were purchased from Tengxin Co. (Chongqing, China). The tumor xenograft model was established by inoculating MDA-MB-231 cells (1*10^6^) mixed with 100 μL of PBS and subcutaneously injected into the around the right lower limb of the mice. The tumors were allowed to grow to ~ 100 mm^3^.

#### In vitro evaluation of the stability and hemolytic capacity

THGP was suspended in various solvents, including DI water, PBS, DMEM and DMEM + 10% FBS, and the changes in the appearance and particle size were monitored. Mouse erythrocytes were acquired, and hemolysis experiments were performed in the presence of HGP and THGP. The hemolysis rate was calculated as described in Additional file 1: Formulation 4 (SI).

#### In vivo assessment of metabolic capacity

Nude mice were randomly divided into various groups (n = 5) and injected with saline or THGP (dose: 40 mg/kg). Blood was sampled from the orbital vein, and the major organs were collected 1, 3, 7, and 14 days after the injection. Serum biochemistry analysis, routine blood tests and H&E staining were performed. A near-infrared fluorescence (NIRF) imaging system (Xenogen IVIS Spectrum; Perkin Elmer, USA) was used for fluorescence imaging (excitation/emission = 748/780 nm). Additional details are described in the Additional file 1: Experimental (SI).

### Tumor visualization

For in vivo US imaging, THGP and HGP (40 mg/kg) were injected into MDA-MB-231 xenograft-bearing nude mice through the tail vein. Two hours later, the tumor was irradiated with LIFU (pulse mode; 1.6 W/cm^2^; 4 min). B-mode and contrast-enhanced ultrasound (CEUS) mode images were acquired using an Esaote ultrasonic diagnostic system (MyLab 90; Italy). The echo intensities of the B-mode and CEUS mode images in each group were quantitatively analyzed using an US image analyzer (DFY-II type; Institute of Ultrasound Imaging, Chongqing Medical University, China), which is an independent research and development product from our laboratory. Additional details for the MRI/PA/US imaging are described in the Additional file 1: Experimental (SI).

### In vivo* evaluation of penetration and synergistic cascade therapy*

Tumor-bearing nude mice were randomly divided into two groups, including DiI-labeled HGP and DiI-labeled THGP, and the corresponding injections were administered as described above. After 2 h, the tumor was removed, fixed with paraformaldehyde and cut into sections. DAPI staining was used to assess the penetration of the nanoplatform in vivo by CLSM.

We determined the effects of synergistic starvation and SDT therapy by assigning nude mice to the following groups (n = 5): saline only, LIFU only, HGP + LIFU, THP + LIFU, TGP + LIFU, and THGP + LIFU. The groups were injected with the same dose of the corresponding compounds (40 mg/kg). In the LIFU-irradiated groups, the tumor was irradiated 2 h after the injection (pulse mode; 1.6 W/cm^2^; 4 min). Mice were treated on days 1, 3, 6, and 9. During the treatment period (16 days), the body weight and tumor size were recorded, and imaging was performed every other day or every four days. Relative tumor volumes were calculated as described in Additional file 1: Formulation 5 (SI). On day 16, the tumors were removed and immediately for H&E staining, TdT-mediated dUTP nick-end labeling (TUNEL), antibodies to proliferating cell nuclear staining antigen (PCNA), and HIF-1α staining. RNA was prepared from the tumor cells for the detection with an Agilent 2100 bioanalyzer (Agilent Technologies, USA) and NanoPhotometer spectrophotometer (Avantor, USA) respectively. The mRNA transcriptome data were used to identify the up/downregulated genes using the filtering criteria (FC ≥ 2.0 (or − 2.0); P < 0.05), and these genes were used for gene ontology (GO) and Kyoto Encyclopedia of Genes and Genomes (KEGG) analyses.

### In vivo* posttreatment biosafety evaluation*

On day 16, blood samples were collected from the orbital vein to detect the levels of alanine aminotransferase (ALT), aspartate aminotransferase (AST), alkaline phosphatase (ALP), urea, creatinine (CREA), and inflammatory cytokines (TNF-α and IL-6). Then, the main organs were collected and stained with H&E.

### Statistical analysis

All data are expressed as the mean ± standard error of the mean. Student's t test was used to determine the statistical significance of the differences between two groups, and the differences between multiple groups were analyzed by two-way analysis of variance (Bonferroni’s correction; *p < 0.05, **p < 0.01, and ***p < 0.001).

## Results and discussion

### ***Physical and chemical properties of ***_***tLyP-1***_***H(Gd)-GOD@PFP***

**Fig. 1:**
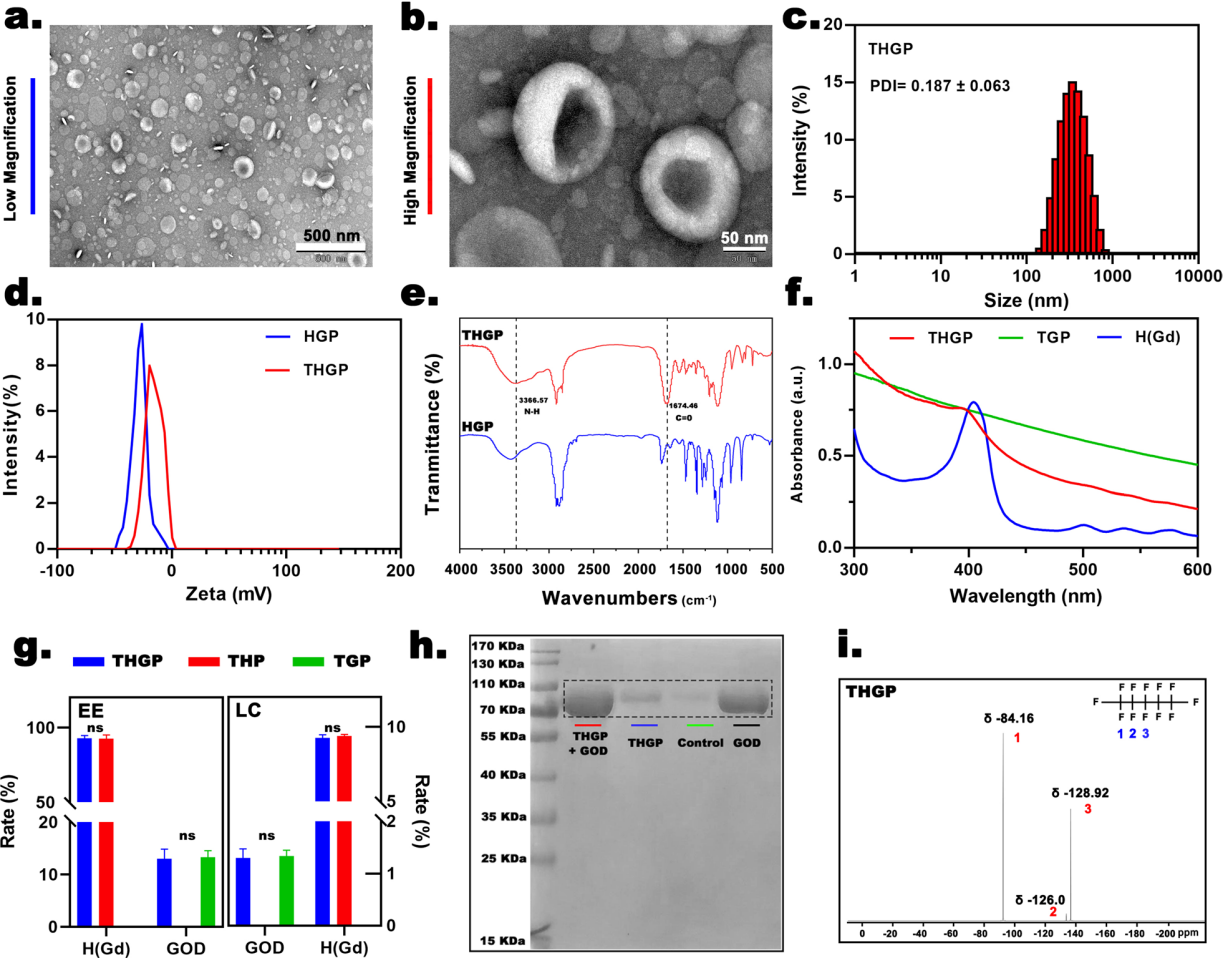
Physical and chemical properties. **a** Representative low- and **b** high-magnification TEM images of THGP. **c** Nanoparticle size distribution and PDI of THGP. **d** Zeta potential and **e** FTIR spectra of HGP and THGP. **f** UV‒VIS spectra of free H(Gd), TGP and THGP. **g** EE and LC of H(Gd) and GOD in various groups. **h** Protein analysis of various groups by SDS‒PAGE. **i** ^19^F-NMR spectra of THGP. H(Gd)-GOD@PFP, _tLyP-1_H(Gd)-GOD@PFP, _tLyP-1_H(Gd)-GOD, _tLyP-1_H(Gd)@PFP and _tLyP-1_GOD@PFP are abbreviated as HGP, THGP, THG, THP and TGP, respectively

The TEM results showed that THGP was spherical and presented a uniform size and clear shell-core structure (Fig. 1a, b). According to the results of a Malvern particle size meter (Fig. 1c), the average diameter of _tLyP-1_H(Gd)-GOD@PFP was approximately 199.13 ± 8.062 nm, and the PDI was approximately 0.187 ± 0.064, indicating that THGP had the size of the nanoplatforms and excellent stability. The short/long-term stability data of THGP under storage conditions are good, and no drastic size change was detected for 2 weeks (Additional file 1: Fig S1a, SI). Due to the positive potential introduced by the tLyP-1 modification, the surface potential of the nanoplatforms changed from − 25.6 ± 0.24 mV (HGP) to − 16.23 ± 0.45 mV (THGP), indicating that modification by tLyP-1 changed the surface charge properties of the nanoplatforms (Fig. 1d). The characteristic N–H and C = O peaks at 3366.47 and 1674.46 cm^−1^ not only confirmed the amination of DSPE-PEG-Mal and tLyP-1 but also indicated the successful fabrication of THGP (Fig. 1e). The results of the FTIR analysis were consistent with a previous study on tLyP-1-based liposomes. [32]. The UV–VIS spectroscopy data (Fig. 1f) showed that the THGP sample retained the absorption peak of H(Gd) at 407 nm, and TGP displayed a smooth UV–VIS spectrum. This result indicated the successful loading of H(Gd), providing a basis for subsequent SDT treatment and MR/PA imaging. The standard curves for H(Gd) and GOD were constructed based on the UV–VIS spectra and HPLC analysis (Additional file 1: Fig. S1b and c, SI). The encapsulation efficiency (EE) and loading capability (LC) of THGP for H(Gd) were 92.7% ± 1.89% and 9.3% ± 0.21%, respectively, and the corresponding values for GOD were 13% ± 1.82% and 1.3% ± 0.17%, indicating that drug loading was ideal for subsequent synergistic therapy (Fig. 1g). Notably, the EE and LC of THP for H(Gd) were 92.7% ± 2.39% and 9.4% ± 0.1%, respectively, and the corresponding values of TGP for GOD were 13.3% ± 1.23% and 1.3% ± 0.12%, respectively, proving that coloading of the nanoplatform with H(Gd) and GOD had no effect on the loading ability of individual compounds. Additionally, a GOD-specific band was present in SDS–PAGE (Fig. 1h), providing evidence that GOD was successfully loaded into THGP. The results of fluorine nuclear magnetic resonance (^19^F-NMR) analysis (Fig. 1i) indicated the presence of a peak at δ -84.16 (6F), which is a typical CF3 peak corresponding to PFP-1. Moreover, the integration and displacement values for δ -126.0, 2F indicated an assignment to intermediate fluorine (PFP-3). The last peak at δ -128.92 (4F) was ascribed to PFP-2. In summary, the results of H(GD), GOD, and PFP coloading and specific tLyP-1 modification indicated that THGP was an ideal means for precise control of the selective execution of the desired activity by a US-unlocked cascade reaction strategy.

### Ultrasound unlocking of the cascade reactions

#### Redox reaction of glucose

**Fig. 2:**
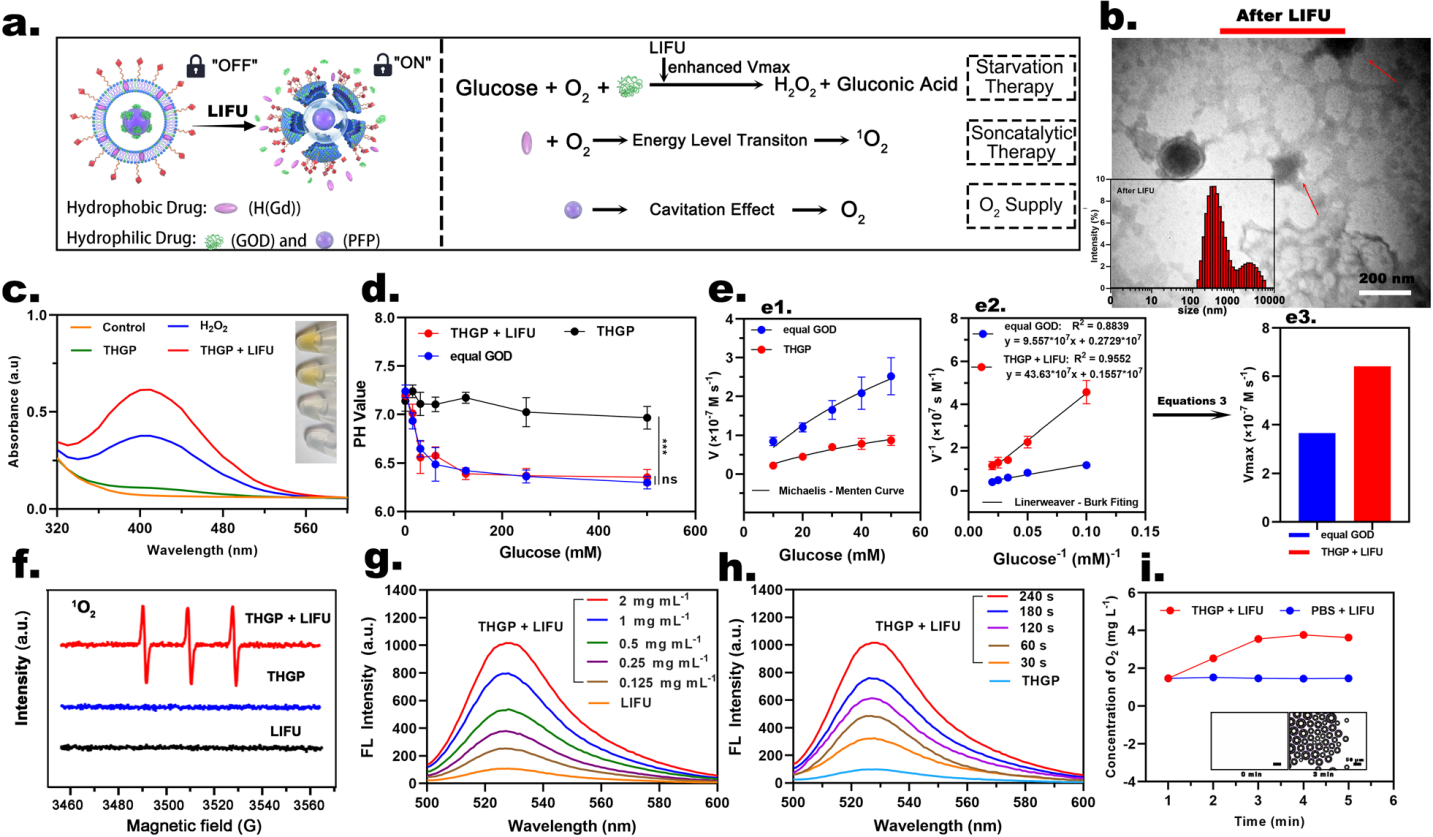
The cascade reactions of THGP. **a** Schematic representation of unlocking the cascade of the reactions of US. **b** TEM images of THGP after LIFU irradiation (inset: the nanoparticle size distributions of THGP after LIFU irradiation). **c** H_2_O_2_ generation in various groups (inset: H_2_O_2_ and titanium sulfate form a yellow titanium peroxide complex). **d** Changes in pH induced by different GOD concentrations (0, 15, 31, 62.5, 125, 250 and 500 mM) (n = 3). **e1** The Michaelis–Menten curve, **e2** the Lineweaver Burk fitting of GOD and THGP with LIFU irradiation for 300 s and **e3** the corresponding Vmax. **f** ESR spectra for THGP with or without LIFU irradiation. **g** SOSG probe signal (Ex/Em = 488/525 nm) showing ^1^O_2_ generation at different THGP concentrations with LIFU irradiation (0, 0.125, 0.25, 0.5, 1 and 2 mg mL^−1^). **h** ^1^O_2_ generation by THGP detected using an SOSG probe after US irradiation for various times (0, 30, 60, 120, 180 and 240 s). **i** O_2_ concentration when THGP or PBS was treated with LIFU for various times (1, 2, 3, 4 and 5 min) (inset: the corresponding optical images were observed under a light microscope at 1 and 3 min)

We verified whether the cascade effect is unlocked by US and checked the performance of the cascade reactions (Fig. 2a). The results of TEM and particle size analysis (Fig. 2b) showed that the integrity of the lipid shell of THGP was destroyed after LIFU irradiation. The nanoparticle size distribution of the nanoplatform was significantly increased to the micron level. This result is consistent with previous ADV effects of nanoultrasonic biomedicine [33]. Therefore, we attributed the underlying mechanism of the US-unlocking cascade reactions to the ADV effect of THGP under LIFU irradiation, which we will discuss in detail in the conclusion of the section that describes US imaging. The results of the H_2_O_2_ detection kit (Fig. 2c) showed that the H_2_O_2_ and THGP + LIFU groups had the same color of the substrate and the same characteristic absorption peak at 415 nm, while the H_2_O and THGP groups did not exhibit these features. Figure 2d demonstrates gluconic acid production through pH value changes. Both free GOD and THGP + LIFU were able to effectively catalyze glucose, and significant differences between the two groups were not detected, confirming that the constructed THGP perfectly retained the redox activity of GOD. In contrast, slight changes were detected in the THGP groups. It is believed that US intervention is a prerequisite for THGP-induced cascade reactions. In addition, in the THGP + LIFU group, the concentration of H_2_O_2_ was increased concomitant to an increase in the concentration of the glucose substrates. When the concentration of the glucose substrate reached 100 mM, H_2_O_2_ generation was limited due to the limited loading of THGP with GOD (Additional file 1: Fig. S2, SI).

Surprisingly, by measuring a series of initial velocities, performing calculations **(**Additional file 1: Formulations 3, SI) and fitting to the Beer-Lambert law and a Michaelis–Menten curve, the relationship between Vmax of redox reaction and irradiation time was determined (Fig. 2e). The Vmax in the THGP + LIFU group was 6.423 * 10–7 M/s, which was significantly better than that of the free equal-GOD groups (3.664 * 10^–7^ M/s) owing to the oscillation and cavitation effects of US. In addition, the Vmax of the THGP group is negligible upon failure to apply LIFU. The Vmax of the THGP + LIFU and free equal-GOD groups showed an apparent LIFU irradiation time-dependent phenomenon (Additional file 1: Fig. S3, SI). Since the entire process is performed at low temperatures. This result suggests that LIFU-induced thermal effects could be excluded, and the cavitation and oscillatory effects induced by LIFU caused rapid and enhanced redox reactions. These results revealed that US unlocking can not only precisely control the selective execution of the desired activity but also further enhance the redox enzymology effect of GOD, which is an exciting result for enhanced synergistic treatment through cascade reactions.

#### Catalytic reaction

Although deprivation therapy can consume glucose while producing H_2_O_2_ in tumors, synergistic therapeutic effects are difficult to achieve due to the weak ROS-dependent toxic effect of H_2_O_2_. Therefore, SDT was used to compensate for this defect. H(Gd) is a metalloporphyrins that undergoes an energy level transition under sound energy to stimulate the valence change and finally catalytically release excess of ROS, especially ^1^O_2_. [34, 35] The ESR spectra results (Fig. 2f) and the absorption peak intensity of SOSG (Fig. 2g and Additional file 1: Fig. S4a, SI) were increased under LIFU irradiation concomitant to an increase in THGP concentration. In contrast, ^1^O_2_ is not produced in solution without THGP, even in the presence of LIFU irradiation. In addition, at the same concentration of THGP, the intensity of the SOSG signal at 525 nm was increased concomitant to an increase in the duration of LIFU irradiation (Fig. 2h and Additional file 1: Fig. S4b, SI). These results not only confirmed that US unlocking is a key step in the cascade effect but also provided evidence for SDT therapy in vivo.

#### O_2_ supply

PFC are the carriers for O_2_ dissolution/delivery, and the solubility of O_2_ in a PFC is up to an order of magnitude higher than that in water. Therefore, during US-activated synergistic therapy, the ADV effect stimulated the release of O_2_ from the PFC, according to the results obtained using a portable oxygen meter (Fig. 2i and Additional file 1: Fig. S5a, SI). Comparison with the THGP and H2O + LIFU groups indicated that the THGP + LIFU group generated a higher O_2_ concentration, while the O_2_ concentration in the THGP + LIFU group was positively correlated with the duration of LIFU irradiation. Notably, after 3 min of LIFU irradiation, the O_2_ concentration reached a plateau, and a large number of micron-sized bubbles were observed. Apparently, these data can be explained by the feedback of the ADV effect. The O_2_ concentration in the THGP + LIFU group was positively correlated with the nanoplatform concentration (Additional file 1: Fig. S5b, SI). Therefore, PFP-loaded THGP is expected to enhance the efficacy of synergistic starvation and catalytic therapy through a US-controllable supply of O_2_.

### Cell experiments

#### Cellular internalization and permeability

**Fig. 3:**
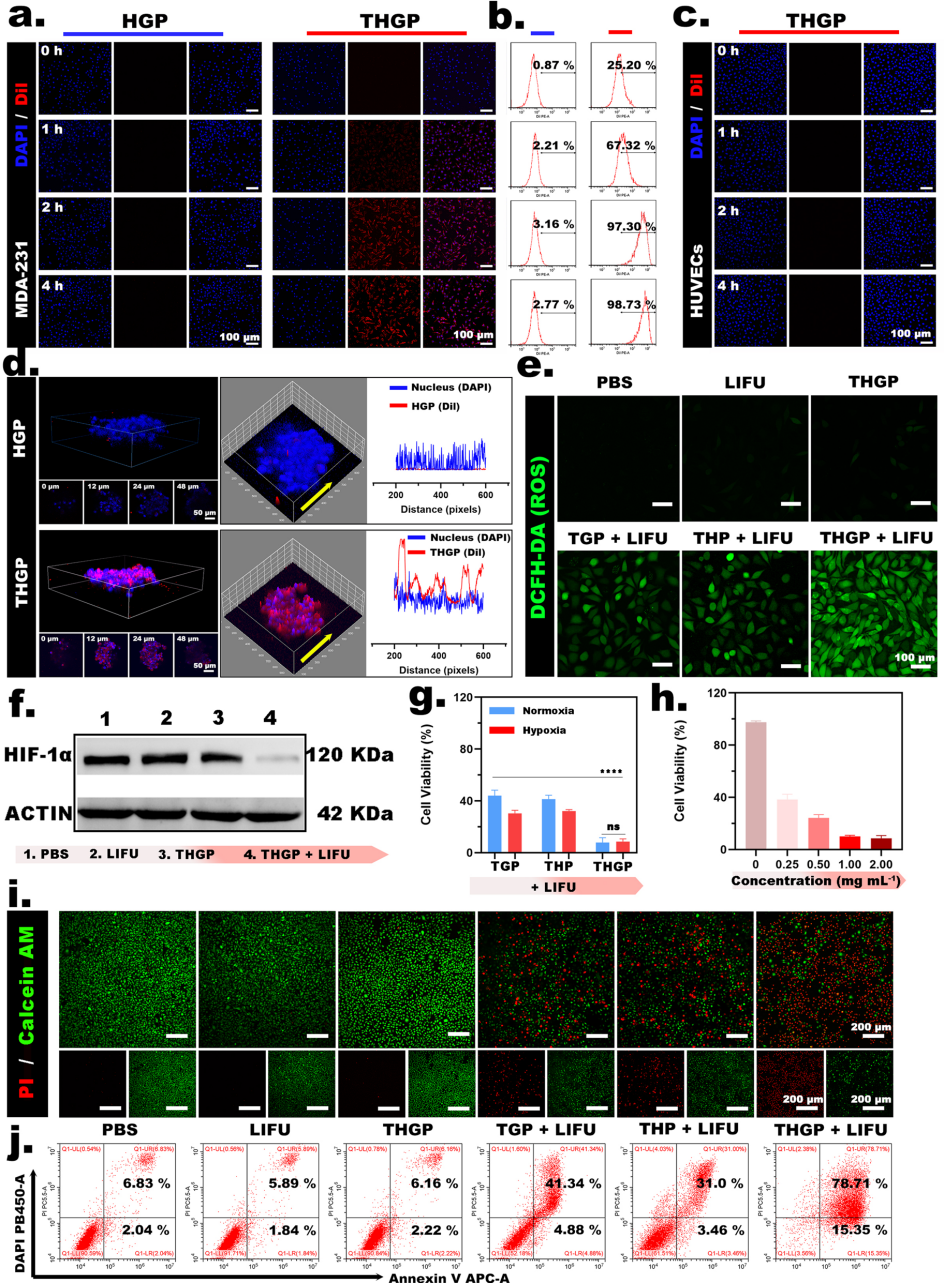
Cell experiment. **a** CLSM images of the internalization of THGP or HGP labeled with Dil in MDA-MB-231 cells after respective incubation at various time points (1, 2, 3 and 4 h). **b** Corresponding FCM analysis. **c** CLSM images of the internalization of THGP labeled with Dil into HUVECs at various time points (1, 2, 3 and 4 h). **d** 3D-CLSM images of THGP or HGP labeled with Dil permeability into MDA-MB-231 cells and the corresponding depth display (0, 12, 24 and 48 μm) (the fluorescence distribution and intensity from the edge to the center of the 3D tumor sphere at a depth of 24 μm). **e** CLSM images of MDA-MB-231 cells after various treatments and subsequently stained with the ROS fluorescence probe DCFH-DA. **f** WB analysis of HIF-1α and ACTIN expression after various treatments (PBS, LIFU, THGP and THGP + LIFU). **g** Relative viabilities of the MDA-MB-231 cells in the TGP + LIFU, THP + LIFU and THGP + LIFU groups in normoxic/hypoxic environments (n = 3). **h** Relative viability of the cells in the THGP + LIFU group with various nanoplatform concentrations (0, 0.25, 0.5, 1 and 2 mg mL^−1^) (n = 3). **i** CLSM images of MDA-MB-231 cells stained with PI (red, dead cells) and calcein-AM (green, live cells) after different treatments. **j** Data of an apoptosis assay performed by FCM

The tLyP-1 peptide contains a tumor target domain and a tissue penetration domain. Therefore, the modification of the nanoplatform with tLyP-1 should be able to overcome the limitations imposed by sequential biological barriers against specific tumor selection and further tissue penetration. CLSM results showed that DiI-labeled THGP infiltrated the cytoplasm and aggregated around the cell membrane in MDA-MB-231 cells (Fig. 3a). The data of FCM (Fig. 3b) showed that the number of DiI-labeled THGP around the cell membrane and into the cytoplasm of MDA-MB-231 cells was higher than that of unmodified HGP used under the same conditions. Interestingly, the level of fluorescence was increased in a time-dependent manner and reached a plateau after 2 h. During the following 2–4 h, the signal remained stable at a high level of fluorescence. This result clearly illustrated the successful modification by the tLyP-1 peptide and verified the tumor cell-targeting ability of the peptide. HUVECs are not only present in most normal tissues but also have a low affinity for the tLyP-1 peptide. [29] Therefore, there was no significant accumulation of DiI-labeled THGP in HUVECs (Fig. 3c).

In addition, comparison with traditional two-dimensional adherent cells indicates that the morphology and function of isolated cells in a three-dimensional culture are closer to the actual growth state of living cells in animals, including biological barriers of a tumor that limit deep penetration of the nanoplatform. After coincubation with DiI-labeled THGP, the tumor spheres were filled with red fluorescence. Then, a three-dimensional tumor sphere model was reconstructed by CLSM to quantify the penetration depth of THGP. Notably, intense and planar fluorescence was observed at depths of 0–48 μm in the THGP group. However, DiI-labeled HGP was sparsely distributed only on the surfaces of the tumorsphere (Fig. 3d). Moreover, we evaluated the fluorescence distribution and intensity from the edge to the center of the 3D tumor sphere at a depth of 24 μm. This result demonstrated a uniform distribution of THGP inside the tumor sphere. In summary, THGP acquired the ability for deep tumor penetration due to the assistance of the tLyP-1 peptide, and these data verified that the tumor-specific nanoplatform provides a prerequisite for efficient O_2_ supply.

#### Intracellular ROS generation and alleviation of hypoxia

H_2_O_2_/^1^O_2_ are produced by deprivation therapy and SDT. In addition, the O_2_ supply unlocked by US is expected to further enhance the effects of synergistic treatment. As shown in CLSM of DCFH-DA staining (Fig. 5e), the results of the treatment of the control groups with PBS and LIFU proved that control MDA-MB-231 cells and the cells treated with US alone did not produce ROS. THGP alone did not produce ROS because the US-unlocking cascade reactions cannot occur without LIFU irradiation. In the TGP + LIFU group, due to the presence of GOD and PFP, TGP released GOD under LIFU irradiation, catalyzing the production of H_2_O_2_ from glucose and thus enabling ROS detection. Similarly, in the THP + LIFU group, H(Gd) was activated under LIFU irradiation, resulting in the transformation of O_2_ into ^1^O_2_ and ROS detection. Notably, in the presence of PFP, GOD, and H(Gd), the THGP + LIFU group demonstrated stronger ROS generation due to the presence of PFP, which provided an additional O_2_ supply for GOD and H(Gd). The data of fluorescence intensity analysis indicated significant differences between the groups (Additional file 1: Fig. S6, SI). Moreover, the hypoxia-inducible factor 1 (HIF-1) complex has been widely recognized as a primary regulator of the response to hypoxia (low oxygen levels). The HIF-1 gene is turned off at normal O_2_ levels (normoxia) and turned during hypoxia, resulting in an increase in protein expression [36]. The protein expression and corresponding gray values are displayed in data of alphaEaseFC assay (Alpha Innotech) (Fig. 3f and Additional file 1: Fig. S7, SI). Due to the hypoxic conditions, the PBS, LIFU-only and THGP-only groups showed high HIF-1α expression. Notably, due to the US-unlocking cascade effect and the O_2_ supply provided by PFP, the hypoxic environment of the tumor cells was significantly abolished in the THGP + LIFU group. In conclusion, these results proved that unlocking the cascade reactions by US is a feasible approach for enhanced synergistic starvation therapy and SDT.

#### Evaluation of the cytotoxicity of synergistic therapy

The data of CCK-8 **(**Additional file 1: Fig. S8a, SI) showed that in the absence of LIFU treatment-mediated conditions, the nanoplatform groups with various concentration gradients and components had no significant effect on cell viability. These findings suggested that LIFU mediation was a prerequisite for the unlocking of the cascade effect.

Next, we applied LIFU irradiation to each group (Additional file 1: Fig. S8b, SI). The results observed in all groups were similar to the data on intracellular ROS generation. The cell survival rate in the THGP + LIFU group was 8.56 ± 1.68%, and this rate was significantly better than that in the THP + LIFU (30.36 ± 1.99%) and TGP + LIFU (32.14 ± 0.89%) groups. Comparison with the HGP + LIFU (94.89 ± 0.89%) group indicated that cellular activity in the THGP + LIFU group was significantly lower. Interestingly, Fig. 3g shows that the cell survival rate in the synergistic treatment group was higher than that in the monotherapy group regardless of the state of cell culture, and there was no difference in the cytotoxicity in the synergistic treatment group. The results on the effect of PFP on O_2_ supply indicated that PFP enhanced the level of oxygen under hypoxic conditions. The cell survival rate in the THGP + LIFU group was clearly concentration-dependent (Fig. 3h).

Similarly, Fig. 3i shows that the number of dead cells in the THP + LIFU, TGP + LIFU, and THGP + LIFU groups was significantly greater than that in the other groups. The number of dead cells in the THGP + LIFU group was considerably greater than that in the other two groups. The results of FCM (Fig. 3j) also showed that the total apoptosis rate in the THGP + LIFU group (early apoptosis: 15.35% and late apoptosis: 78.71%) was significantly higher than in the THP + LIFU (early apoptosis: 3.46% and late apoptosis: 31%) and TGP + LIFU groups (early apoptosis: 4.88% and late apoptosis: 41.34%). Therefore, all data on the cell survival rate proved that synergistic starvation therapy and SDT acting via US-unlocked cascade reactions are feasible at the cellular level.

### Biosafety evaluation

**Fig. 4:**
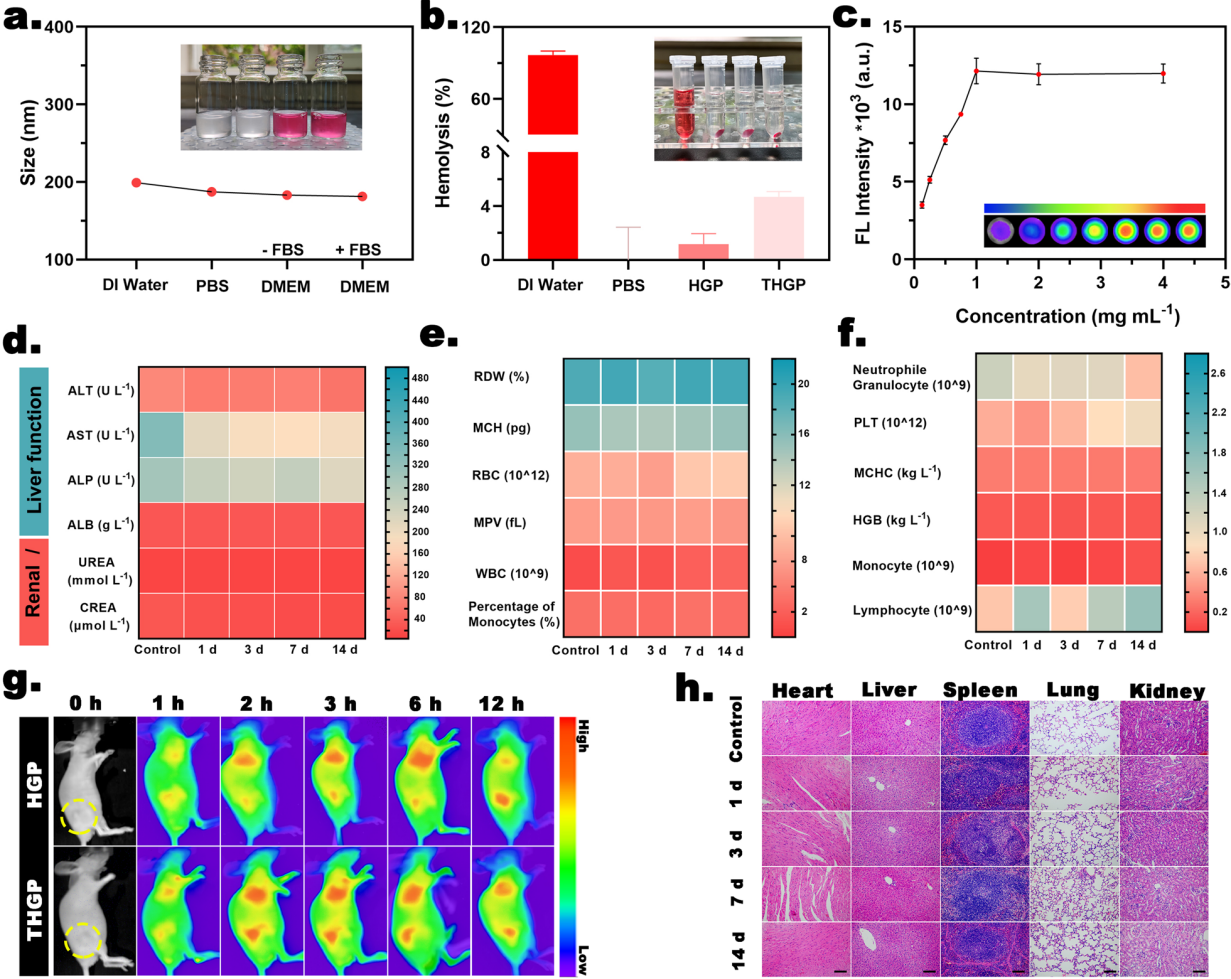
Biosafety evaluation. **a** The mean size of THGP in various suspensions (inset: corresponding digital images). **b** Hemolysis analysis of HGP and THGP (inset: corresponding digital images) (n = 3). **c** In vitro fluorescence imaging intensity of DIR-labeled THGP (inset: corresponding fluorescence images) (n = 3). **d** and **e** Routine blood analyses on various days after intravenous injection of THGP (n = 5). (**f**) Blood biochemical analyses of the blood on various days after intravenous injection of THGP (n = 5). **g** In vivo metabolic fluorescence images after intravenous injection with THGP and HGP for various time intervals. **h** H&E staining of major organs on various days after intravenous injection of THGP (scale bar: 200 μm)

Before conducting in vivo experiments, according to the requirements of the animal ethics guidelines, we fully evaluated the safety and metabolic characteristics of the nanoplatform to avoid unnecessary sacrifice. Figure 4a shows that THGP had good solubility in various solutions, and supersaturated precipitation or aggregation was not observed. The results of hemolysis experiments (Fig. 4b) showed that THGP and HGP did not have hemolytic properties, and the corresponding hemolysis rates were lower than an international standard (5%). These results indicated that it was possible to further evaluate the safety of the nanoplatform in vivo after intravenous injection.

The results of routine blood analysis (Fig. 4d and e) from day 1 to day 14 after THGP administration via intravenous injection showed no acute (1 and 3 days), short-term (7 days), or long-term (14 days) hematotoxicity. Figure 4f also shows the lack of that there was no significant differences in hematological parameters, hepatic function (ALT, AST, ALP, and ALB) or renal indicators (UREA and CREA) between the control and THGP groups. Finally, H&E staining of the main organs (heart, liver, spleen, lung, and kidney) after intravenous injection showed that each group had little effect on healthy organs (Fig. 4h).

The biosafety of THGP was more comprehensively demonstrated by evaluating the biological distribution and metabolic characteristics of the nanoplatform. The concentration-dependent capability of THGP-based fluorescence imaging was shown using a DIR-labeled nanoplatform. When the concentration of DIR-labeled THGP reached 1 mg mL^−1^, the fluorescence intensity reached the upper limit of detection of the instrument (Fig. 4c). After labeled THGP was injected into the tail vein, the distribution of fluorescence was detected at the tumor site from 1 to 12 h, and the strongest fluorescence intensity was detected at 2 h. However, 2 h after the injection of HGP, slight fluorescence aggregation was detected, indicating that THGP exhibited enhanced tumor recognition and aggregation ability due to the assistance of the tLyP-1 peptide (Fig. 4g and Additional file 1: S9a, SI). Twelve hours after intravenous injection of THGP, fluorescence imaging of isolated significant organs (heart, liver, spleen, lung, and kidney) in the majority of multiple mice showed strong tumor aggregation (Additional file 1: Fig. S9b, SI). These results demonstrated that the nanoplatform modified with the tLyP-1 peptide had good biosafety and tumor-specific selectivity, which was beneficial for subsequent tumor visualization and in vivo antitumor treatment.

### Tumor visualization

#### PA and MR imaging

**Fig. 5:**
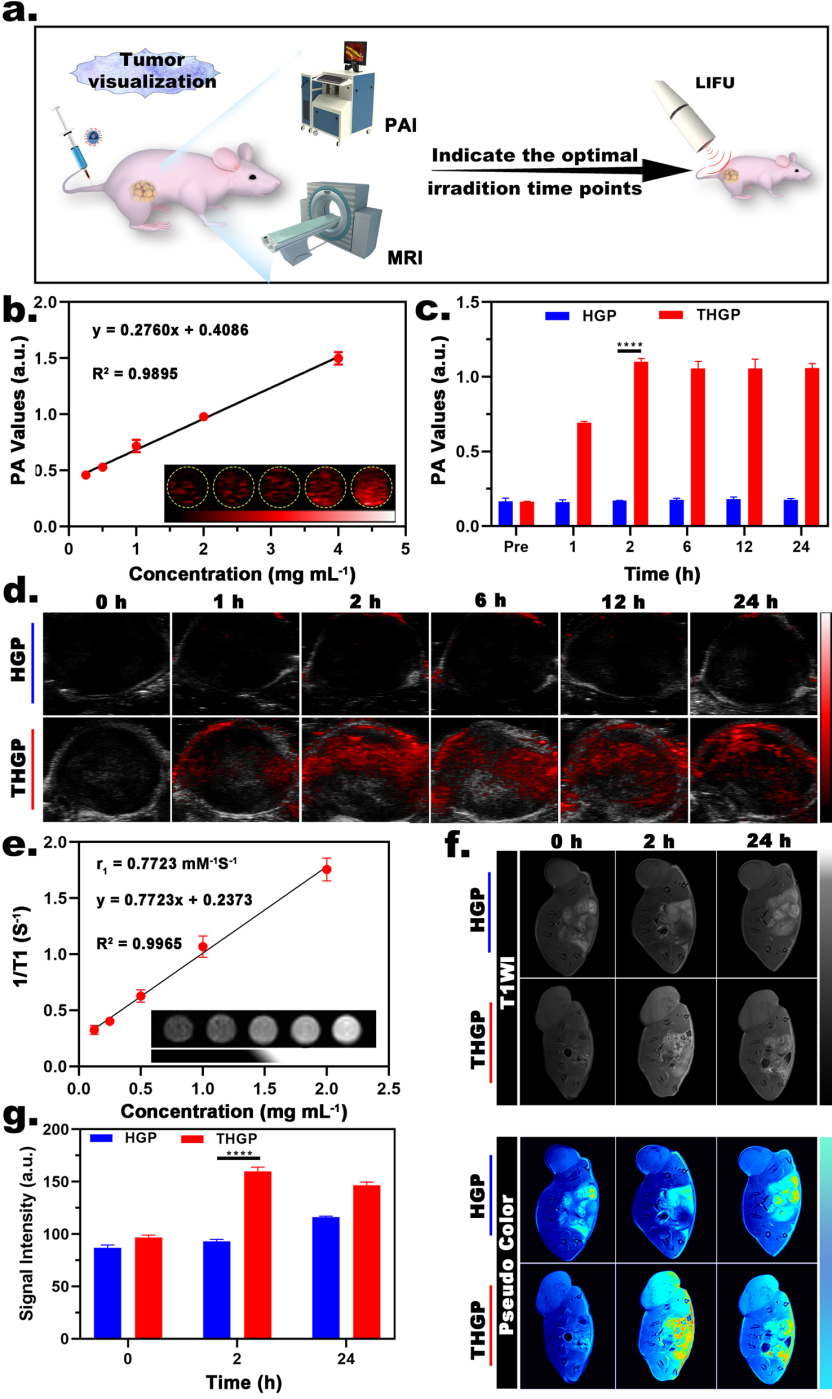
PA and MR imaging. **a** Illustration of tumor visualization. **b** In vitro PAI of THGP at various concentrations (inset: corresponding PA images). **c** In vivo PA values and **d** corresponding PA images after intravenous injection of THGP or HGP (n = 3). **e** In vitro MRI of THGP at various concentrations (inset: corresponding MR images). **f** MR images and **g** corresponding signal intensities after intravenous injection of THGP or HGP (n = 3)

Noninvasive visual imaging was applied to obtain real-time feedback for the detection of time points at an optimal aggregation concentration of the nanoplatform in solid tumors, providing a sufficient and reasonable basis for subsequent selection of the time points for LIFU irradiation (Fig. 5a). Previous studies demonstrated a strong light absorption capability of H(Gd) at 710 nm; thus, THGP can convert a short-pulse laser to a PA signal under these conditions. [37] Fig. 5b shows that the PA signal intensity increased with increasing THGP concentration. The data from subsequent in vivo experiments (Fig. 5c, d) demonstrated the lack of PA signal in the tumor area before the injection. However, 1 h after the injection, a PA signal began to appear in the THGP group; the signal peaked at 2 h and continued to be detectable until 24 h. However, there was no significant difference in the PA signal intensity in the tumor area before and after the injection in the HGP group.

Similarly, magnetic resonance imaging (MRI) of THGP depended on the superior magnetic relaxation ability of Gd. As shown in Fig. 5e, the T1-weighted signal of MRI showed a noticeable concentration-dependent effect, and the value of the relaxivity of THGP was 0.7723 mM^−1^S^−1^. The tumor area showed a low signal in both groups that was similar to the signal in the surrounding soft tissue. After the injection of THGP, the signal in the tumor area became bright and enhanced at 1 h, peaked at 2 h, and continued to 24 h. However, HGP injection did not induce significant changes in the signal of the tumor area (Fig. 5f, g). The data of MRI were similar to the results of a previous PA imaging assay, indicating that THGP achieved tumor-specific aggregation that reached a maximum at 2 h due to the assistance of tLyP-1. In particular, real-time visualization of the changes in the concentration of exogenous molecules inside solid tumors was needed, and these assays were crucial for subsequent controllable O_2_ supply experiments performed in the present study.

#### US imaging

**Fig. 6:**
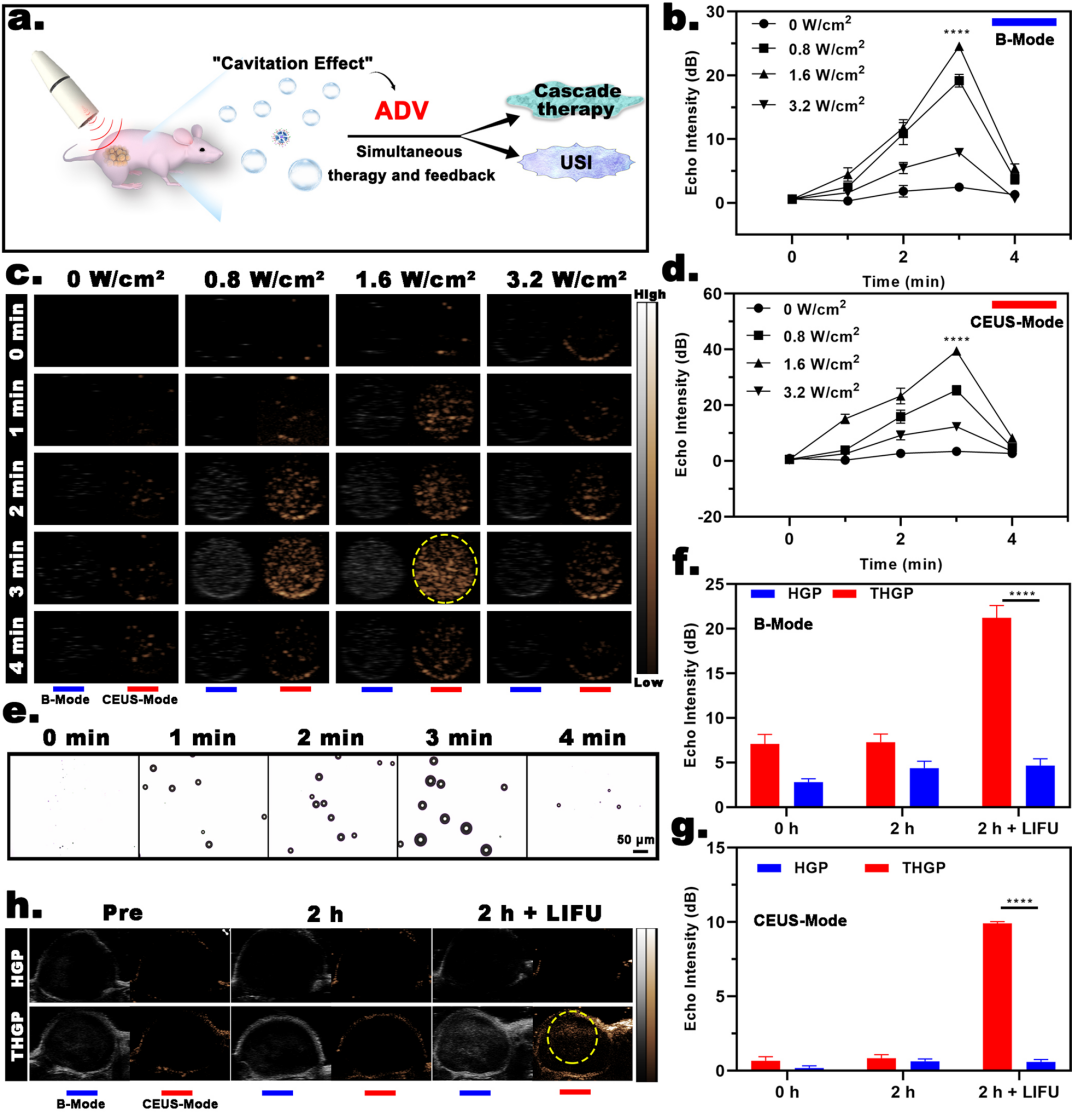
US imaging. **a** Illustration of the ADV process of USI. **b** In vitro echo intensity in B-mode (n = 3), **d** echo intensity in CEUS-mode (n = 3); and **c** the corresponding US images when THGP was treated with different LIFU-time and power conditions. **e** Optical microscopy images of the ADV process when THGP was treated with LIFU. **f** In vivo echo intensity in the B-mode (n = 3); **g** echo intensity in CEUS-mode (n = 3); and **h** the corresponding US images 2 h after intravenous injection with THGP or HGP. The tumors were exposed to LIFU

Because PFP-based nanodroplets can be used as medical biomaterials for O_2_ supply and generate cavitation effects under US stimulation to realize the potential of US contrast imaging, we speculated that the cascade reaction is unlocked by US due to the nanoultrasonic biomedicine-based ADV effect. Notably, the ADV effect can be visualized by US imaging feedback (Fig. 6a). Therefore, the use of US molecular imaging to verify the ADV effect and evaluate the influence of different LIFU parameters on the ADV effect is of great significance for a combination of ultrasonic medicine and nanoplatform-based cascade engineering. Because free PFP or PBS does not undergo a liquid‒gas phase transition (27), PFP-loaded THGP is the critical factor in achieving US imaging due to the ADV effect. The echo images in the B-mode and CEUS-mode in all groups demonstrate an increase in the signal intensity concomitant to an extension of irradiation time at the same processing power; the echo images were the strongest when irradiation time was 3 min, and the echo images of the control group showed no significant changes. When the power was varied, an increase in power induced an upward trend in the echo signal in the B-mode and CEUS-mode in all groups, and the echo signal at 1.6 W/cm^2^ was the strongest (Fig. 6c). Figure 6b and d also show that the echo signals of THGP in the B-mode and CEUS-mode were the strongest 3 min after the application of LIFU (1.6 W/cm^2^). Quantitative analysis showed that the echo signal reached a maximum value of 39.42 dB in the strongest CEUS-mode group**.** Notably, when the irradiation duration was longer than 3 min or the irradiation power was higher than 1.6 W/cm^2^, the echo signal sharply dropped. We speculated that UTMD caused by the ADV effect induced the bursting of a large number of microbubbles.

Next, light microscopy was used to demonstrate that the size of THGP was LIFU irradiated (Fig. 6e), indicating that the liquid PFP core was transformed into a gas state upon acoustic stimulation and that nanoscale THGP expanded to micron-scale bubbles, which was consistent with the results on O_2_ release. Interestingly, the number of micron-scale bubbles gradually decreased after 4 min of LIFU irradiation, which may result of further expansion of oversized bubbles causing their collapse. According to the theory of ADV, nanoscale THGP not only realized US imaging but also realized drug release in the process of expansion to rupture, and these events appear to be the key factor responsible for the simultaneous emergence of US-unlocked cascade reactions and US-dependent visualization.

In the case of US imaging in vivo (Fig. 6h), the tumor tissue showed regular hypoechogenicity before the injection of THGP. However, there were no significant differences in the US imaging of the tumor region for 2 h after the injection. According to the results of feedback of PA and MR imaging, this time point should have been the best for evaluation of THGP aggregation. Therefore, when the tumor was irradiated with LIFU for 4 min, the tumor tissue injected with THGP showed clearly enhanced US images in B-mode and CEUS-mode. In contrast, there was no detectable echo signal in the HGP + LIFU group. Moreover, quantitative analysis by an US image analyzer was consistent with the data of US imaging (Fig. 6f, g). These results proved that LIFU addition at the best targeting time point more efficiently and accurately induced the occurrence of ADV to better realize US-controlled O_2_ supply and cascade treatment. In brief, the visualization features of the nanoplatform, pointed out the biggest differences between THGP and the agents used in previous studies, i.e., the biological potential of nanoultrasonic medicine can be used to achieve a controllable cascade reaction, and more importantly, the unlocking process can be visualized by US imaging as feedback.

### In vivo* evaluation of penetration and synergistic cascade therapy*

**Fig. 7:**
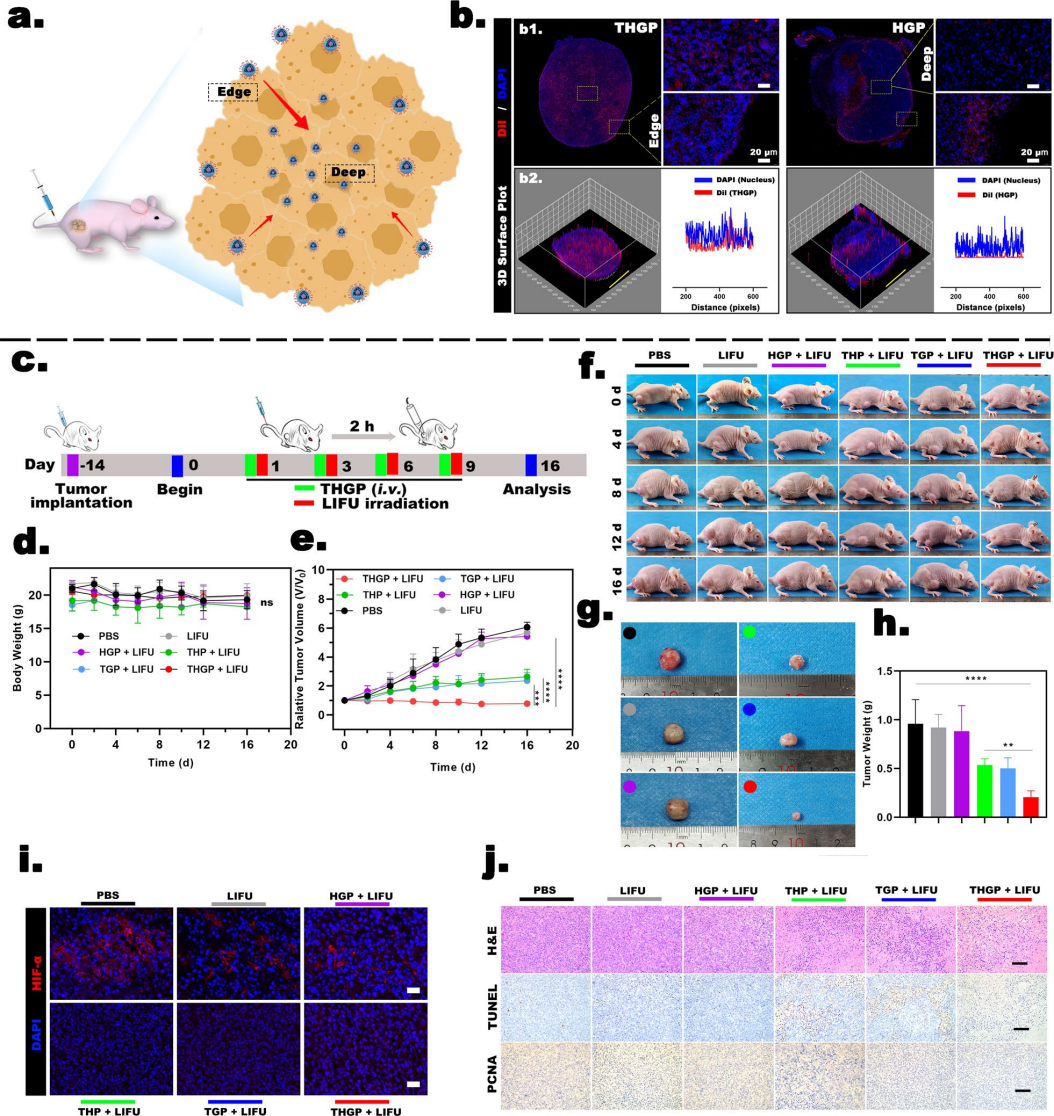
Penetration and synergistic therapy. **a** Illustration of the tumor penetration ability of the nanoplatform. **b1** CLSM images of the nanoplatform permeating the tumors in MDA-MB-231 tumor-bearing mice and **b2** the corresponding 3D reconstruction surface plot after intravenous injection of THGP or HGP. **c** Schematic illustration of MDA-MB-231 cell tumor xenograft establishment, PBS and various nanoplatfrom administration modalities, and analysis. **d** Body weight curves of mice in various groups during the treatment process (n = 5). **e** Relative tumor volumes of mice in the various groups during the treatment (n = 5). **f** Representative digital images of MDA-MB-231 tumor-bearing mice after various treatments. **g** Representative digital images of excised tumors after various treatments and **h** the corresponding tumor weight curves (n = 5). **i** Immunofluorescence images of mouse tumors stained for HIF-1α (red, hypoxia level) and with DAPI (blue, nuclei) after various treatments (scale bar: 200 µm). **j** H&E, TUNEL, and PCNA staining of the tumor tissues of various treatment groups (scale bar: 200 µm)

We continued evaluating the permeability of the nanoplatform in vivo (Fig. 7a). The fluorescence distribution images of the whole isolated tumor in the THGP group (Fig. 7b1) showed that DiI-labeled THGP (red fluorescence), due to the assistance of the tLyP-1 peptide, was evenly distributed from the edge to deep regions of the solid tumor. In contrast, in the HGP group, red fluorescence was present only at the tumor margins and did not penetrate deep inside. This phenomenon was due to the fact that macromolecular substances of a specific size are more likely to penetrate into the tumor tissue and are retained for a long time, and this phenomenon also known as the enhanced permeability and retention (EPR) effect. [38] However, the EPR effect is currently applicable only to animal tumor models, and a good EPR effect has not been demonstrated in preclinical experiments. The results of the 3D reconstruction surface plot (Fig. 7b2) showed that red fluorescence at a depth from 200 μm to 600 μm inside the tumor was significantly stronger in the THGP group than in the HGP group. Therefore, the tLyP-1-modified nanoplatform represents a more rational design to achieve an efficient O_2_ supply.

We then designed a reasonable treatment scheme to better evaluate the antitumor ability of synergistic cascade therapy (Fig. 7c). After 16 days of observation and recording, there were no significant differences in body weight or physiological behavior between the groups (Fig. 7d). First, we proved that the experimental scheme did not affect the normal physiological activities of mice. Second, the data on the relative tumor volume in the treatment groups showed that the results of antitumor treatment in vivo were consistent with the data on cytotoxicity (Fig. 7e). Specifically, the saline- and LIFU-only groups were used as the control groups, and LIFU irradiation did not produce mechanical stress or thermal effects that interfered with the evaluation of the outcomes of treatment. The HGP + LIFU group did not show a therapeutic effect. In combination with the results of cellular experiments, these findings further demonstrated that the visualized nanoplatform cannot achieve a synergistic antitumor effect without modification by the tLyP-1 peptide even after LIFU intervention because the nanoplatform does not have tumor specificity and cannot overcome the biological barriers of the tumors. Notably, the relative tumor volume after 16 days was changed 0.79 ± 0.15-fold in the THGP + LIFU group, and the relative tumor volumes in the THP + LIFU and TGP + LIFU groups were changed 2.37 ± 0.5-fold and 2.63 ± 0.46-fold, respectively. This result illustrated that GOD- and H(Gd)-loaded THGP, starvation treatment, and SDT unlocked by US achieved a synergistic treatment effect as expected. At the same time, representative digital photos of mice during the treatment (Fig. 7f), digital images of excised tumors after the treatment (Fig. 7g), and corresponding tumor weight curve results after the treatments (Fig. 7h) indicated that tumor growth was significantly inhibited in the THGP + LIFU group due to synergistic cascade therapy.

To further confirm that the loading of PFP is not only a prerequisite for realization of the US-unlocked cascade reactions but can also provide for O_2_ synergy to synergistic therapy, we evaluated the amelioration of hypoxic state of the tumor by an assay of the expression of HIF-1α (Fig. 7i). Markedly enhanced tumor hypoxic environment in the THGP + LIFU, THP + LIFU, and TGP + LIFU groups was ameliorated by PFP loading. More clear results of internal in the tumors were obtained by immunohistochemical analysis of isolated tumors. A large area of apoptotic features, such as nuclear lysis and cytoplasmic compactness in the tumor, appeared in the H&E-stained sections of the THGP + LIFU group, clearly showing a significant decrease in tumor cell staining compared with other groups. Moreover, deep staining of PCNA (the proliferation index) was decreased and the expression of TUNEL (the apoptosis index) was increased in the THGP + LIFU group (Fig. 7j).

**Fig. 8:**
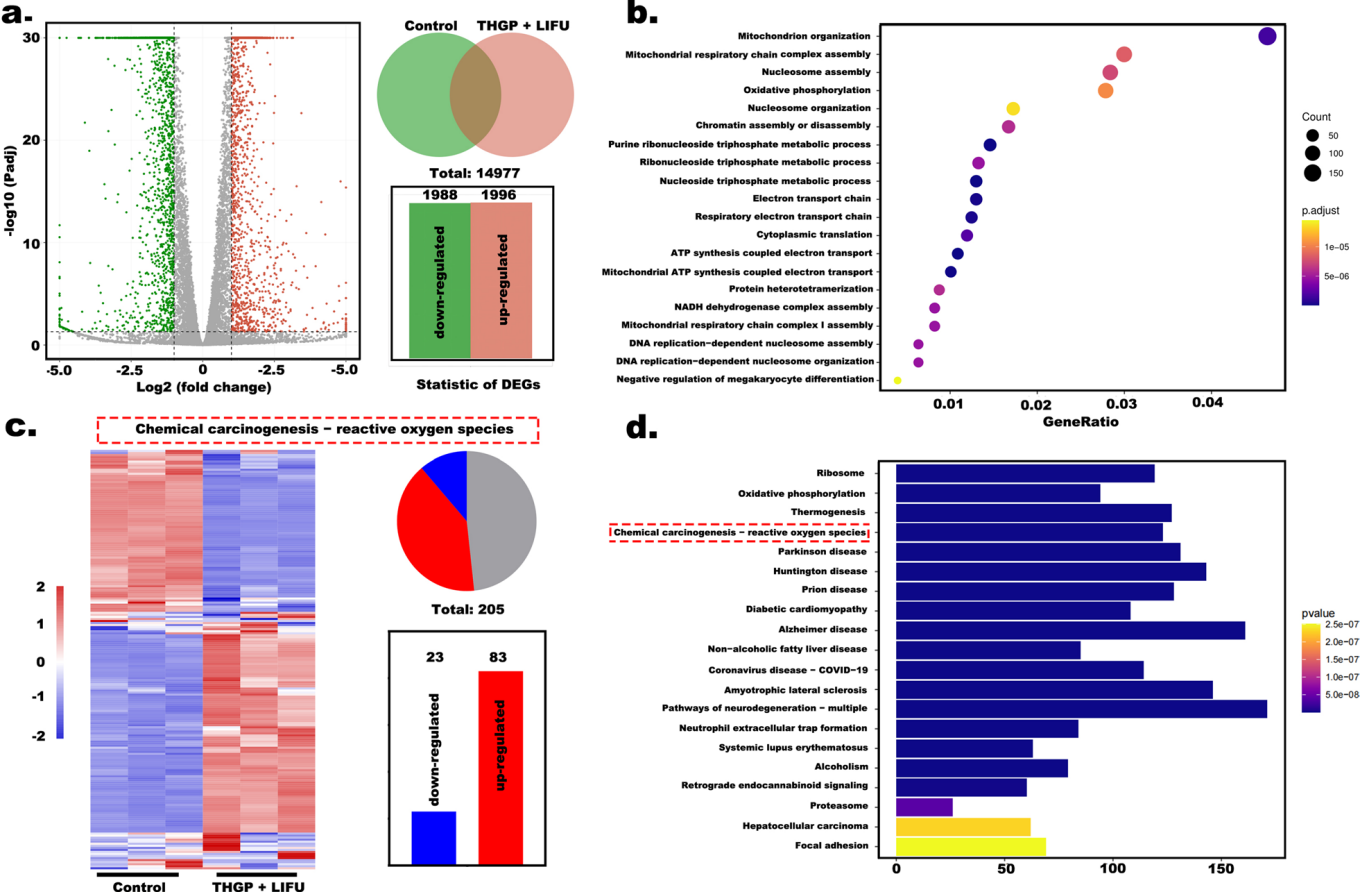
Transcriptomic analysis. **a** Venn diagram analysis of differentially expressed genes in the control and THGP + LIFU groups. **b** Top 20 differentially expressed genes according to the data of GO enrichment. **c** Venn diagram analysis and heatmap of the differentially expressed genes of the chemical carcinogenesis-ROS pathway genes. **d** Top 20 differentially expressed genes by KEGG pathway enrichment analyses

In addition, the results of gene sequencing in the control and THGP + LIFU groups indicated good intragroup consistency and significant intergroup differences (Additional file 1: Fig. S10a and b, SI). A total of 14,977 mRNA transcripts were detected, including 1,996 upregulated and 1,988 downregulated genes (Fig. 8a). Related biological processes according to GO illustrated that synergistic therapy induced significant differences in the expression of energy expenditure-related proteins, including ATP synthesis-coupled electron transport, oxidative phosphorylation, and mitochondrial respiratory chain complex assembly (Fig. 8b). The results obtained using KEGG pathway analysis of the top 20 significantly differentially expressed genes indicated that the chemical carcinogenesis-ROS pathway was associated with synergistic therapy and was activated after THGP + LIFU treatment (Fig. 8d). This pathway (Fig. 8c) included a total of 205 differentially expressed mRNAs, corresponding to 23 upregulated and 83 downregulated genes. The results of gene enrichment analysis suggested a significant discrepancy. These results not only illustrate the realization of cascade therapy but also deepen our understanding of nanoplatform therapy achieved through an analysis of differentially expressed genes.

### In vivo evaluation of posttreatment biosafety

**Fig. 9:**
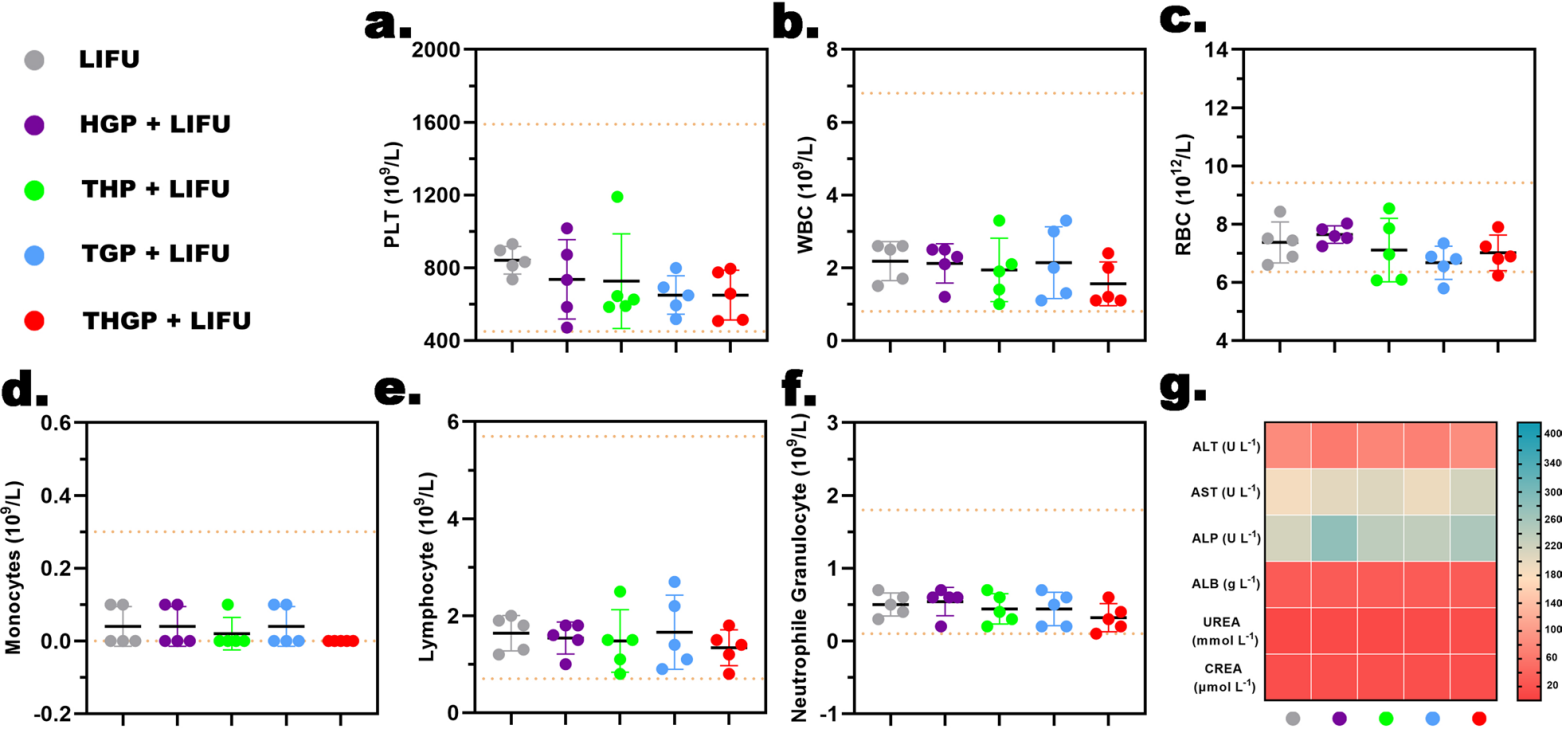
Posttreatment biosafety. (**a**-**f**) Routine blood examination of MDA-MB-231 tumor-bearing mice 16 days after administration of various treatments (inset dotted lines: upper and lower limits of the normal range) (n = 5). **g** Biochemical analysis of the blood of MDA-MB-231 tumor-bearing mice 16 days after administration of various treatments, including: hepatic function (ALT, AST, ALP, ALB) or renal indicators (UREA, CREA)

There were no significant differences in the blood samples at 16 days after LIFU irradiation, including routine blood analysis, hepatic function, renal function, and levels of inflammatory cytokines (Fig. 9a–g, Additional file 1: Fig. S11a and b, SI). The results of H&E staining of major organs after 16 days of treatment showed a lack of major organ damage and toxic side effects (Additional file 1: Fig. S11c, SI). These data confirmed that the treatment had a good antitumor effect and excellent biological safety.

## Conclusions

In summary, we successfully developed a tumor-penetrating visualizable nanoplatform that can overcome the current therapeutic dilemma of nanoplatform engineering, including off-target effects and the uncontrolled release of O_2_ supply, through a cascade of reactions unlocked by US. Modification with the surface-functionalized tLyP-1 peptide, the nanoplatform with the ability to specifically and efficiently aggregate into MBA-MB-231 tumors in vivo and in vitro. The optimal aggregation time was determined by visualization imaging (MR/PA). The liquid–gas phase transition of internal PFP was mediated by LIFU to achieve ADV so that the nanoplatform precisely released GOD, H(Gd), and PFP and to enable USI at the tumor site. Furthermore, this nanoplatform activated the functions of the following components precisely mediated by LIFU: GOD consumes O_2_ and glucose through an enzymatic redox reaction, generates ROS (H_2_O_2_) and gluconic acid, and realizes starvation therapy; H(Gd) consumes O_2_ and produces ROS (^1^O_2_) through energy level transition; and the O_2_ affinity and carrying capacity of PFP can alleviate tumor hypoxia in situ, providing efficient O_2_-dependent therapy. Importantly, the study preliminarily confirmed that LIFU can promote the redox reactions of GOD rather than the thermal effect of LIFU. Therefore, the cascade enhancement effect of THGP mediated by US is not only a simple superposition of starvation and sonodynamic treatment but also a clever design for the specific microenvironment of malignant tumors (hypoxic state, redox-sensitive state, and high-level glucose consumption). This approach not only realizes efficient and synergistic double treatment driven by O_2_ dependency but also demonstrates US unlocking through visual imaging, which is of prospective significance for cross-fusion studies combining nanoplatform-based cascade engineering with ultrasonic medicine.

We discussed in detail the changes in the concentrations of exogenous molecules (nanoplatform aggregation and ADV effect) detected by imaging. However, the changes in the concentrations of endogenous molecules, including oxyhemoglobin and deoxyhemoglobin, etc., was not evaluated in the present study. Therefore, we will conduct additional detailed studies in the future to determine the effect of O_2_ supply on the changes in the concentrations of endogenous molecules.

## Supplementary Information

**Additional file 1: Fig S1. **(a) Nanoparticle size distributio of THGP in PBS solutions serum at different time points. (b) Standard curves of H(Gd) and (c) GOD. **Fig S2. **Generated H_2_O_2_ of different concentration of glucose substrate. **Fig S3. **The relationship between Vmax of redox reaction and irradiation time (0, 60, 120, 180, 240 and 300 s). **Fig S4.** (a) The absorption peak intensity of SOSG in different concentration (0, 0.125, 0.25, 0.5, 1 and 2 mg mL^-1^). (b) The absorption peak intensity of SOSG in different LIFU irradiation times (0, 30, 60, 120, 180 and 240 s). **Fig S5. **(a) Concentration of O_2_ in different treatment groups. (b) Concentration of O_2_ of the THGP with different concentration. **Fig S6. **FL intensity of ROS produced in different treatment groups. **Fig S7. **Relative gray value of different treatment groups. **Fig S8.** (a) Relative cell viability of TGP, THP and THGP groups with different concentrations. (b) Relative cell viability of different treatment groups. **Fig S9.** (a) In vivo metabolic FL intensity after intravenous injection with THGP and HGP for various time intervals. (b) FL imaging of excised major organs and tumors. **Fig S10.** (a) Pearson correlation analysis. (b) PCA dimensionality reduction analysis. **Fig S11.** (a) and (b) Elisa of the levels of inflammatory cytokines of MDA-MB-231 tumor-bearing mice 16 days after administration of various treatments, including: TNF-α and IL-6. (c) H&E staining of the major organs 16 d after different treatments were administered.
