# Supplementary material for: Tumor-penetrating nanoplatform with ultrasound “unlocking” for cascade synergistic therapy and visual feedback under hypoxia
Source: J Nanobiotechnology. 2023 Jan 25;21:30. doi: 10.1186/s12951-023-01765-x (PMC9878980; doi:10.1186/s12951-023-01765-x)
Supplement: Supplementary file 1 — Additional file 1: Fig S1. (a) Nanoparticle size distributio of THGP in PBS solutions serum at different time points. (b) Standard curves of H(Gd) and (c) GOD. Fig S2. Generated H2O2 of different concentration of glucose substrate. Fig S3. The relationship between Vmax of redox reaction and irradiation time (0, 60, 120, 180, 240 and 300 s). Fig S4. (a) The absorption peak intensity of SOSG in different concentration (0, 0.125, 0.25, 0.5, 1 and 2 mg mL-1). (b) The absorption peak intensity of SOSG in different LIFU irradiation times (0, 30, 60, 120, 180 and 240 s). Fig S5. (a) Concentration of O2 in different treatment groups. (b) Concentration of O2 of the THGP with different concentration. Fig S6. FL intensity of ROS produced in different treatment groups. Fig S7. Relative gray value of different treatment groups. Fig S8. (a) Relative cell viability of TGP, THP and THGP groups with different concentrations. (b) Relative cell viability of different treatment groups. Fig S9. (a) In vivo metabolic FL intensity after intravenous injection with THGP and HGP for various time intervals. (b) FL imaging of excised major organs and tumors. Fig S10. (a) Pearson correlation analysis. (b) PCA dimensionality reduction analysis. Fig S11. (a) and (b) Elisa of the levels of inflammatory cytokines of MDA-MB-231 tumor-bearing mice 16 days after administration of various treatments, including: TNF-α and IL-6. (c) H&E staining of the major organs 16 d after different treatments were administered. [file 12951_2023_1765_MOESM1_ESM.docx]

**Additional file**

**Tumor-penetrating nanoplatform with ultrasound“unlocking”for cascade synergistic therapy and visual feedback under hypoxia**

*Zhuoyan Xie^1, 2#^, Junrui Wang^2, 3#^, Yuanli Luo^2^, Bin Qiao^2^, Weixi Jiang^2^, Leilei Zhu^1,2^, Haitao Ran^2^, Zhigang Wang^2^, Wei Zhu^5*^, Jianli Ren^2^**^*^ and Zhiyi Zhou^2,4*^*

1. *Department of Ultrasound, Chongqing General Hospital, Chongqing 401147, China.*
2. *Chongqing Key Laboratory of Ultrasound Molecular Imaging, The Second Affiliated Hospital of Chongqing Medical University, Chongqing 400010, China.*
3. *Department of Radiology, The Second Affiliated Hospital of Chongqing Medical University,*

*Chongqing 400010, China.*

1. *Department of General Practice, Chongqing General Hospital, Chongqing 401147, China.*
2. *Department of Medical College, Hubei University for Nationalities, Enshi Hubei Province 445000, China.*

**1. Additional file experiment**

**1.1. Synthesis of the _tLyP-1_H(Gd)-GOD@PFP**

Firstly, the mixture of DSPE-PEG_3400_-tLyP-1 (4 mg), DPPC (8 mg), DPPG (2 mg), cholesterol (2 mg) and H(Gd) (2 mg) were dissolved in 8 mL of chloroform and the solution was transferred into a round flask to form lipid films by a rotary evaporator in 50 ℃ water bath for 1 h. After that, 5 mL of deionized (DI) water was added to the flask to hydrate the lipid films for further use. Next, 200 μL of GOD aqueous solution (10 mg mL^-1^) and 200 μL of PFP were mixed together and emulsified with an acoustic vibrator (Heat System Inc, USA) for 2 min (ON 5 s, OFF 5 s) in an ice bath as the first-step emulsion. And then, the lipid films were added to the GOD-PFP mixture and emulsified for another 6 min (ON 5 s, OFF 5 s) as the second-step emulsion. Finally, the liposomes were purified by centrifugation (8000 rpm, 5 min) for three times and stored at 4°C for further use. H(Gd)-GOD@PFP were prepared with the same way expect for changing DSPE-PEG_3400_-tLyP-1 into DSPE-PEG_3400_. Similarly, _tLyP-1_H(Gd)@PFP or _tLyP-1_H(Gd)-GOD was prepared without GOD or PFP mixed in the first-step emulsion. _tLyP-1_GOD@PFP were prepared without H(Gd) in the step of rotary evaporation. Fluorescently labeled nanoplatform only need to add corresponding dyes (DiI, DiR) to chloroform during rotary evaporation.

**1.2. Ultrasound unlocking of the cascade reactions**

To avoid the thermal effect of LIFU, the above treatment was carried out in ice water at 4 °C. Then, the irradiated solution was continuously monitored with an enzyme labeling instrument (10 min, 30 s intervals) to calculate the velocities. The fitting curve was plotted with the glucose concentration as the abscissa and the velocity as the ordinate. The calculation method is described in **Additional file formulation 3** (**SI**). According to the previous calculation method, the maximum velocities (Vmax values) of each irradiation time were obtained, and the fitting curve was drawn with the processing time as the abscissa and the maximum speed as the ordinate. Furthermore, GOD or THGP (with the same concentration of GOD) was added to different concentrations of glucose (31.25-1000 mg mL^-1^) for treatment with LIFU (pulse mode, 1.6 W/cm^2^, 4 min).

The SOSG probe reacts with ^1^O_2_ from SDT to form an endoperoxide product with green fluorescence. Briefly, 10 μL of SOSG solution was mixed into 2 mL of sample solution with different concentrations of glucose (0-2 mg mL^-1^). The mixtures were irradiated with LIFU (pulse mode, 1.6 W/cm^2^, 4 min).In addition, a 2 mg mL^-1^ sample solution was irradiated with LIFU (pulse mode, 1.6 W/cm^2^; 0-180 s). A fluorescence spectrometer (Cary Eclipse, Agilent Technologies, USA) was used to determine the generation of ROS by measuring the fluorescence intensity.

**1.4. Cell culture**

HUVECs and MDA-MB-231 were both incubated in a humidified atmosphere containing 5 % CO_2_ at 37 °C. MDA-MB-231 cells were grown in DMEM medium supplemented with 10 % FBS (v/v) and 1 % penicillin-streptomycin (v/v). And similarly, HUVEC were cultured in RPMI-1640 supplemented with 10% FBS and 1 % penicillin-streptomycin. Hypoxic conditions only need to replace the gas environment (1% O_2_, 5% CO_2_, 94% N_2_ gas mixture to culture cells in a closed incubator).

**1.4.1. Synergistic therapy cytotoxicity evaluation**

To test the synergistic starvation and sonodynamic therapeutic effect, MDA-MB-231 cells and HUVECs were seeded at a density of 1*10^5^ cells/well in 96-well plates for 24 h. As appropriate according to treatment group (PBS, LIFU, HGP + LIFU, THP + LIFU, TGP + LIFU and THGP + LIFU), nanozymes were resuspended in serum-free medium at the same concentration (2 mg/mL, 100 µL), and LIFU was applied as indicated (pulse mode, 1.6 W/cm^2^, 4 min). After 24 h of incubation, cell viability was detected by CCK-8 assay.

To criticality evaluate the US unlocking ability to determine the cytotoxicity of each nanozyme, MDA-MB-231 cells were incubated with different concentrations (0.25-2 mg/mL) of each nanozyme for 4 h. Cell viability was determined by the CCK-8 method. For qualitative analysis, MDA-MB-231 cells in a confocal dish were treated differently (PBS, LIFU, HGP + LIFU, THP + LIFU, TGP + LIFU and THGP + LIFU) and then irradiated or not with LIFU (pulse mode, 1.6 W/cm^2^, 4 min). All groups were stained with PI (red, dead cells) and calcein-AM (green, surviving cells) and observed by CLSM. For quantitative analysis, MDA-MB-231 cells in a 6-well plate were treated as described above and the apoptosis rates were measured by flow cytometry (FCM; CytoFLEX, Beckman Coulter, USA).

**1.5. PA imaing**

PA signals were collected by excitation light with different wavelengths (680-970 nm), and PA imaging experiments were carried out under optimized excitation light of 710 nm. Different concentrations of the THGP (0.25, 0.5, 1, 2 and 4 mg mL^-1^) were placed in the agarose gel model, and PA imaging was scanned with the excitation wavelength of 710 nm by Vevo LAZR , and PA signals were quantitatively analyzed in the target area. For *in vivo* PA imaging, HGP and THGP (40 mg/kg) were injected into MDA-MB-231 xenograft-bearing nude mice through the tail vein. PA images of the tumor area were obtained at different time points (0, 1, 2, 6, 12 and 24 h postinjection) and the PA signal was quantitatively analyzed.

**1.6. MR imaing**

For *in vivo* MRI, THGP and HGP (40 mg/kg) were injected into MDA-MB-231 xenograft-bearing nude mice chosen at random through the tail vein. Images were obtained at 0, 2 and 24 h after injection. The T1 relaxation rate was calculated by linear fitting of the reciprocal of the T1 (1/T1) relaxation time and Gd concentration (μM). The THGP (0.125, 0.25, 0.5, 1, 2 mg mL^-1^) were diluted with DI water in EP tubes, and then, the T1WI MR imaging was preformed using a 3.0 T MRI machine (MAGNETOM Prisma, Siemens, Germany) with the instrumental parameters setting as follows: repetition time (TR) = 650.00 ms, echo time (TE) =11.00 ms, a section thickness = 2.1 mm.and then 3.0 T MRI machine .

**1.7.** **US imaging**

For *in vitro* US imaging, THGP radiated with different acoustic intensity of LIFU (0, 0.8, 1.6, 3.2 W/cm^2^) and for different total of time (0, 1, 2, 3 and 4 min) were placed in the agarose gel model. Moreover, the THGP were treated with LIFU for 0, 1, 2, 3, 4 min, then the appearance of LDB was observed by light microscopy (Olympus DP70, Canada).

**1.7. Biosafety evalution**

For *in vitro*, different concentrations of DiR-labeled THGP (0.125 - 4 mg mL^-1^) placed in the 96 well plates were imaged and detected quantitatively. For *in vivo*, tumor-bearing nude mice were randomly divided into three groups: DiR-labeled HGP and THGP, which were intravenously injected at a dose of 200 μL respectively. And all FL images were also obtained at 0, 1, 2 and 12 h after injection. After that, the tumor and main organs of THGP group (heart, liver, spleen, lung and kidney) were separated and their fluorescence images were collected.

**2. Additional file formulation**

1. EE (%) = M (Drug encapsulated in liposome) / M (Total drug added) ×100%
2. LC (%) =M (Drug encapsulated in liposome) / M (Total materials) ×100%
3. $\frac{1}{v}$ = $\frac{\mathrm{Km}}{\mathrm{Vmax}}$ ( $\frac{1}{[S ]}$ + $\frac{1}{\mathrm{Km}}$)

Where *v* is the initial velocity, *Vmax* represents the maximum reaction velocity, *[S]* is the

substrate concentration and *Km* expresses the Michael constant.

1. Hemolysis (%) = (Sample absorbance − PBS absorbance) / (DI-water absorbance − PBS group absorbance) × 100%
2. V/V_0_ = (The current volume) / (The volume at the beginning of treatment) ×100%. V= length × width^2^ / 2
3. **Additional file figure**

**
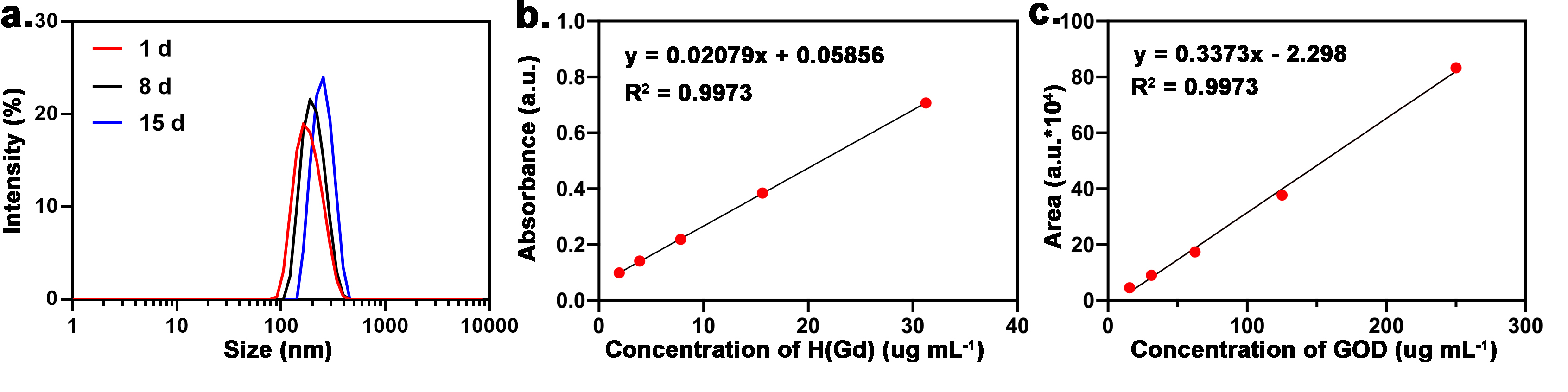
**

**Fig S1.** (a) Nanoparticle size distributio of THGP in PBS solutions serum at different time points. (b) Standard curves of H(Gd) and (c) GOD.


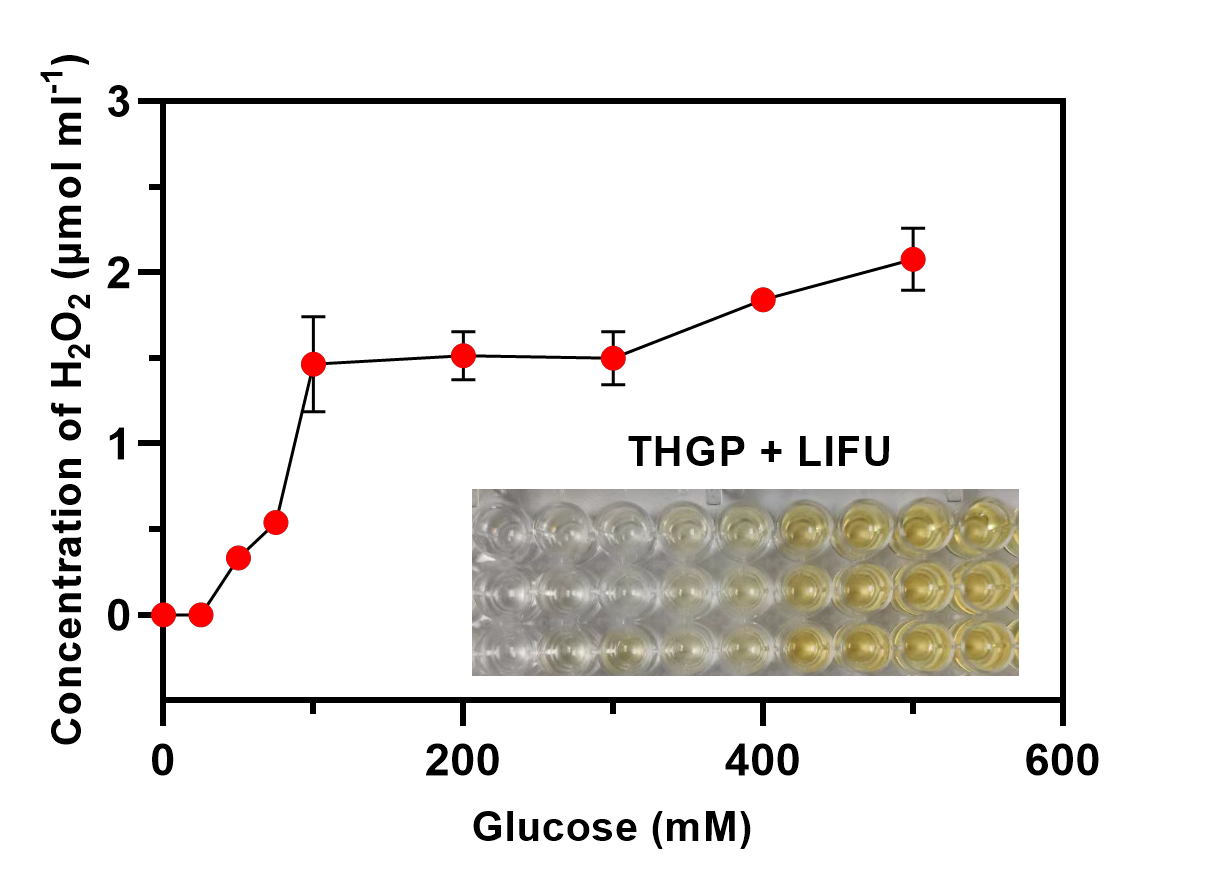


**Fig S2.** Generated H_2_O_2_ of different concentration of glucose substrate.


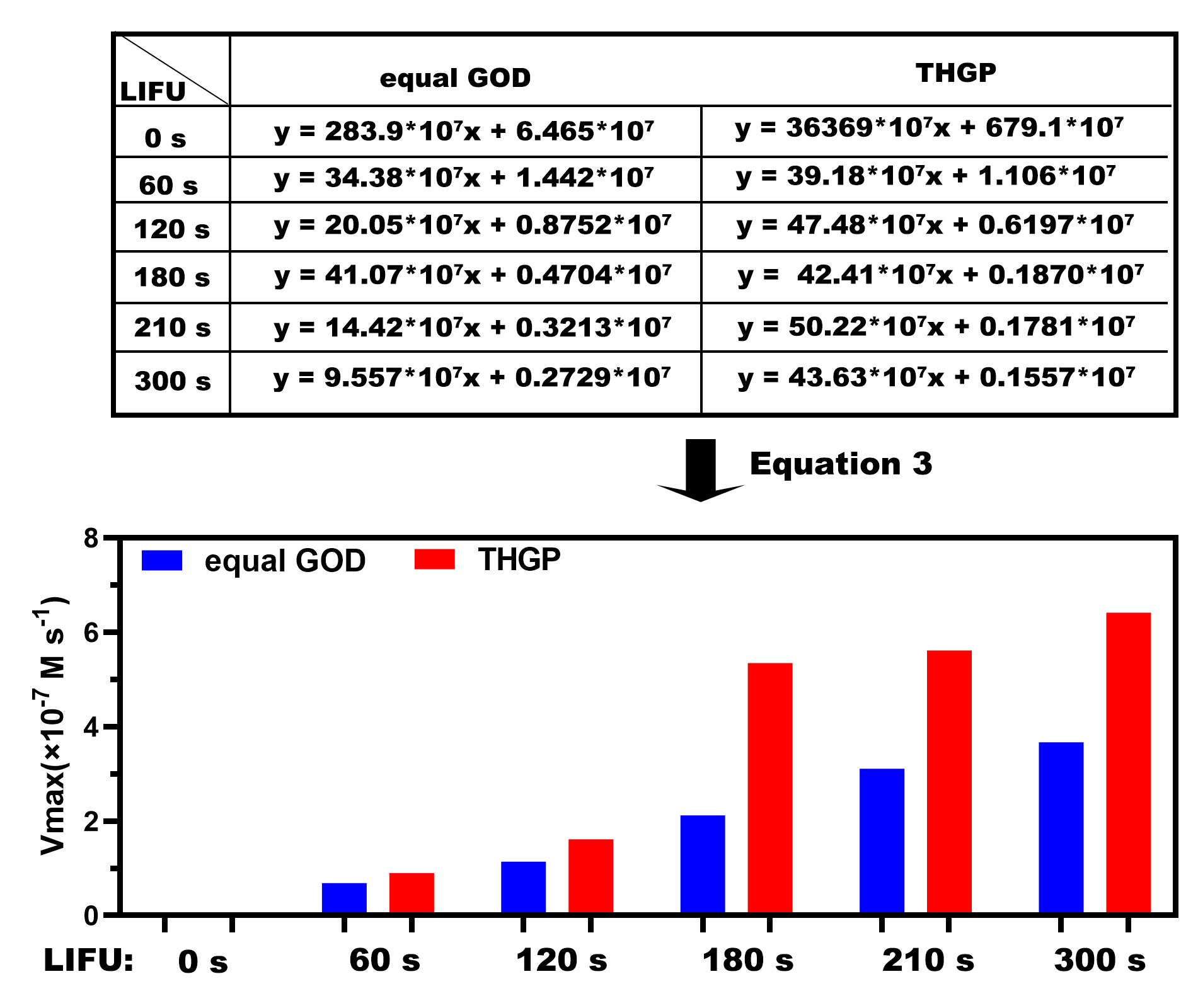


**Fig S3.** The relationship between Vmax of redox reaction and irradiation time (0, 60, 120, 180, 240 and 300 s)


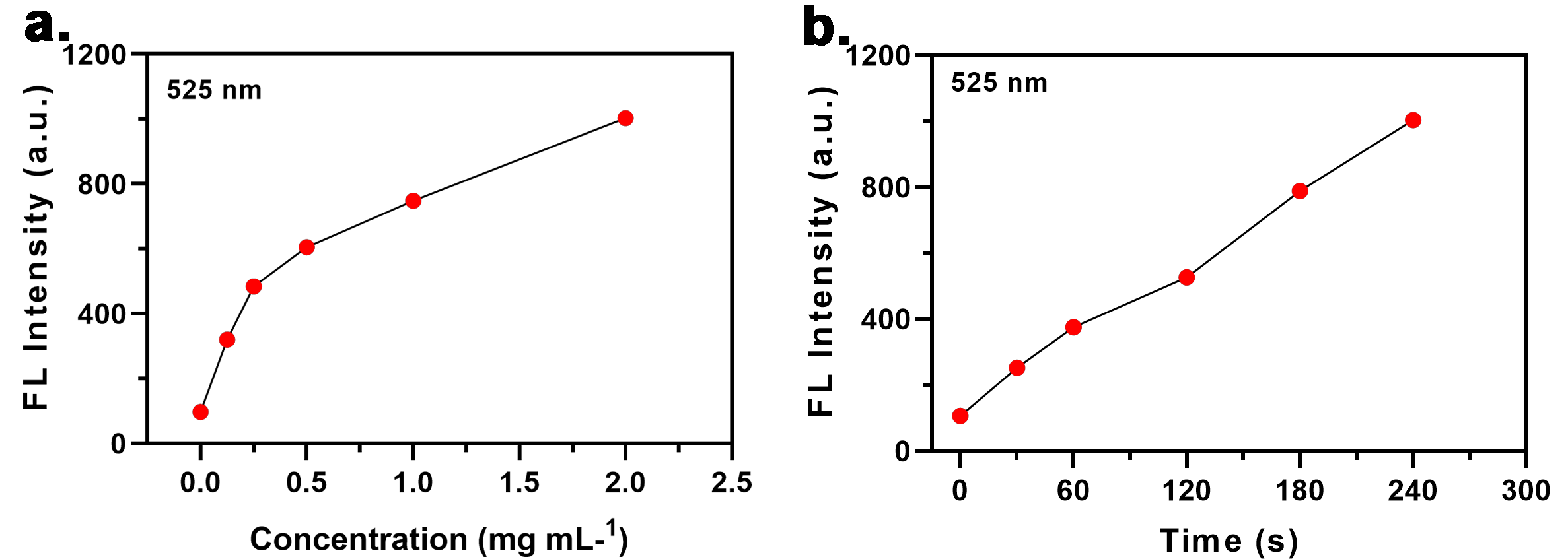


**Fig S4.** (a) The absorption peak intensity of SOSG in different concentration (0, 0.125, 0.25, 0.5, 1 and 2 mg mL^-1^). (b) The absorption peak intensity of SOSG in different LIFU irradiation times (0, 30, 60, 120, 180 and 240 s).


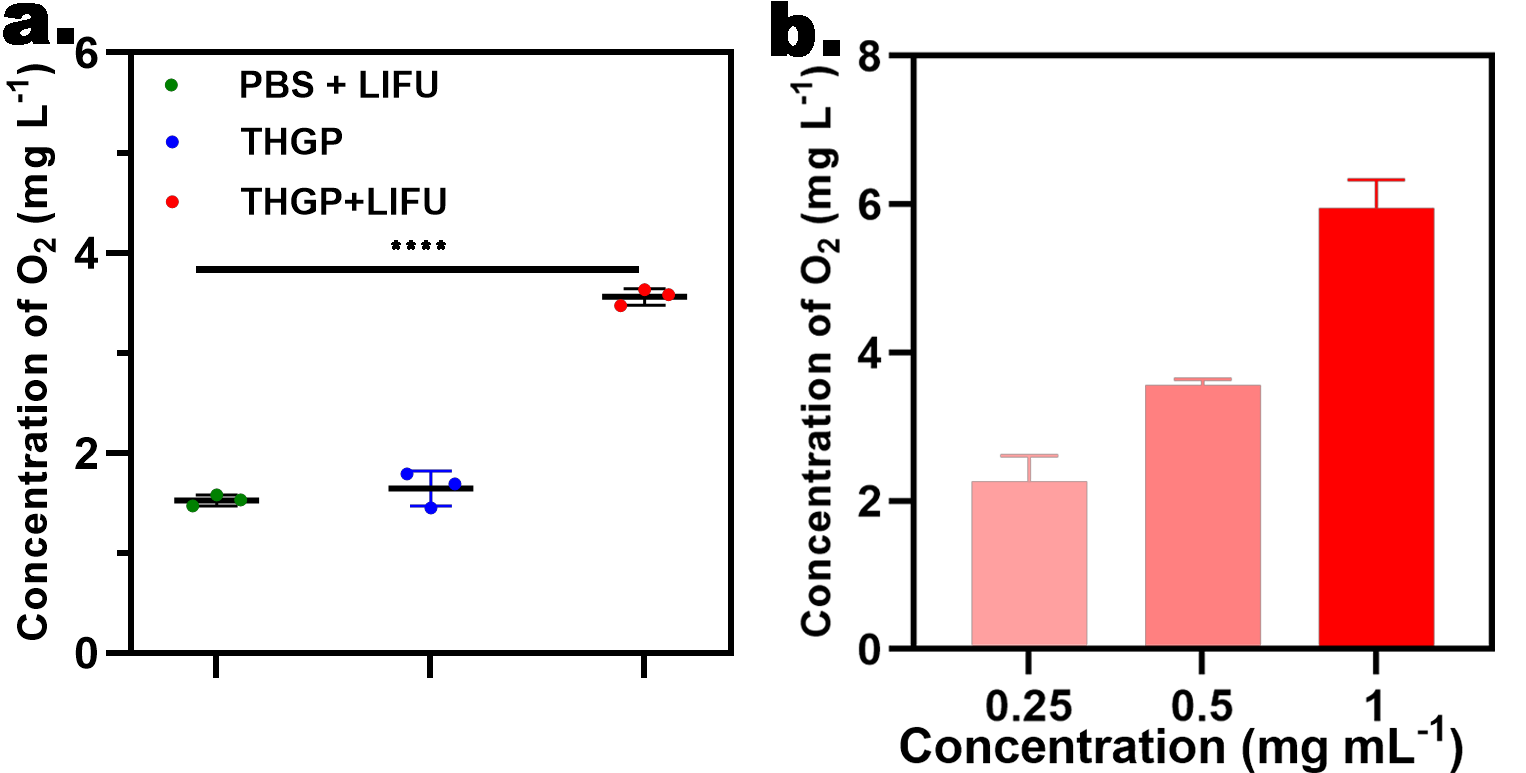


**Fig S5.** (a) Concentration of O_2_ in different treatment groups. (b) Concentration of O_2_ of the THGP with different concentration.


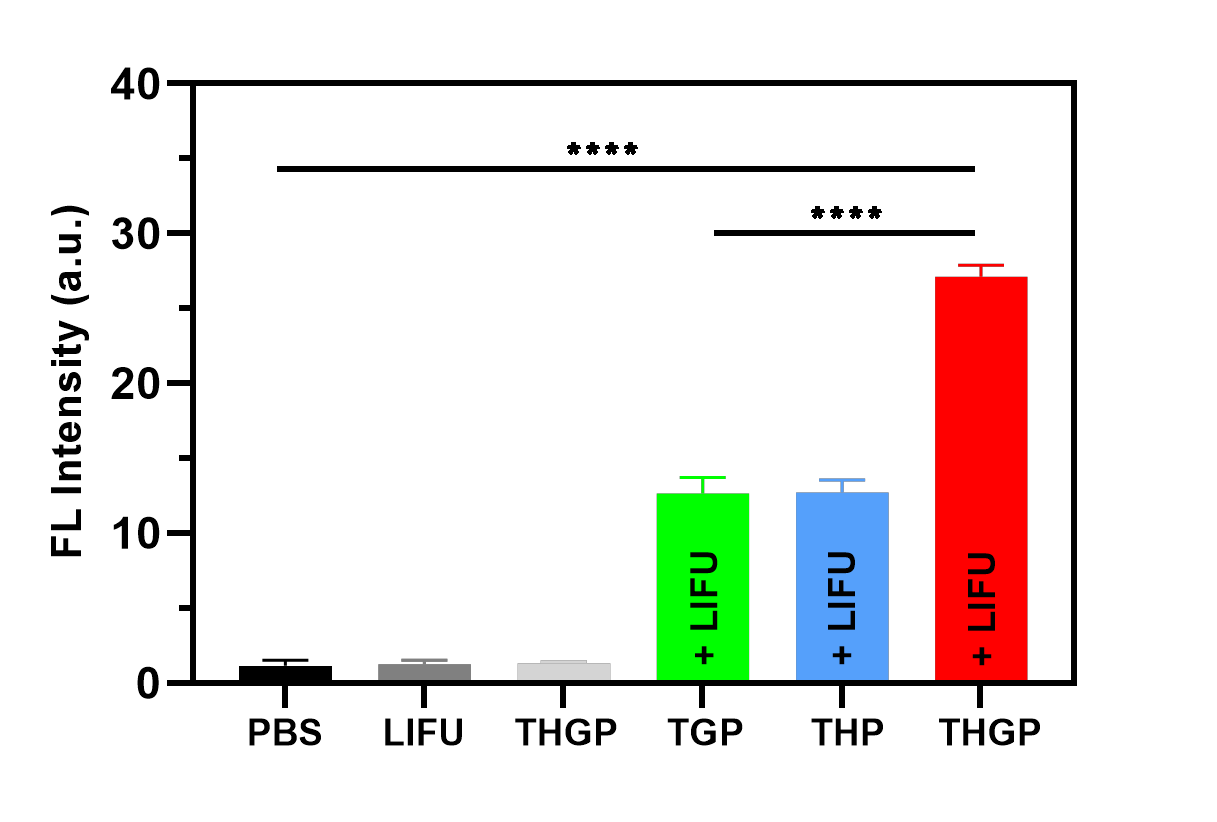


**Fig S6.** FL intensity of ROS produced in different treatment groups.


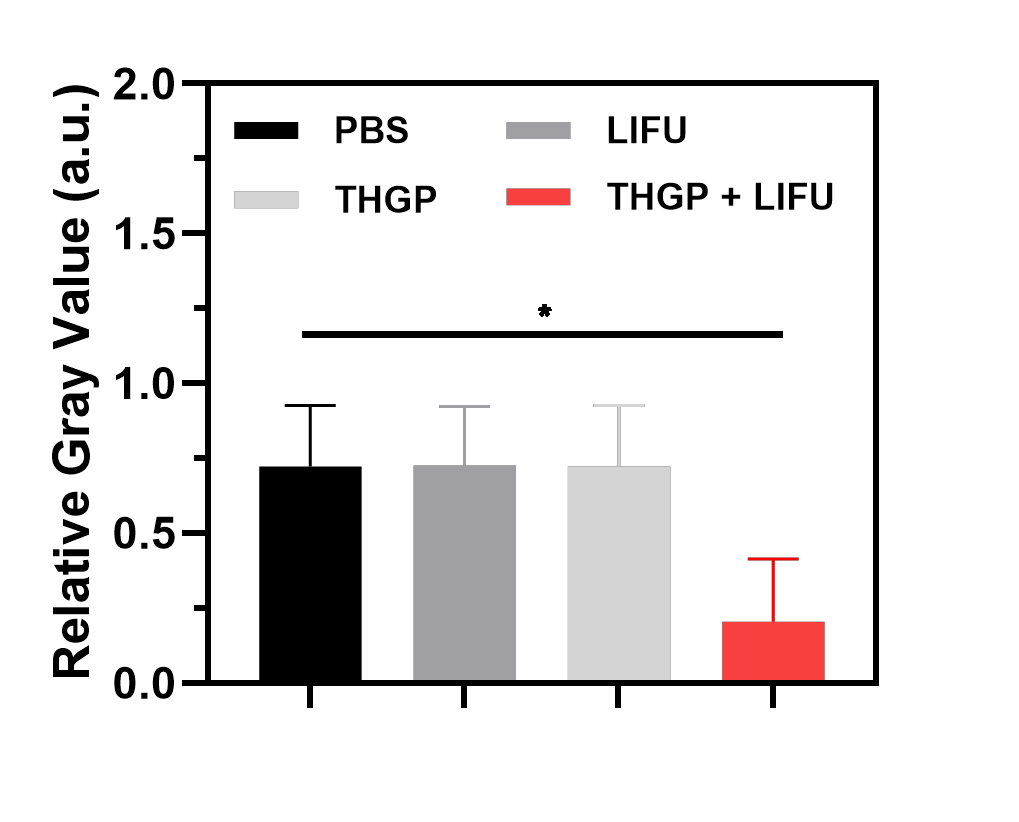


**Fig S7.** Relative gray value of different treatment groups.


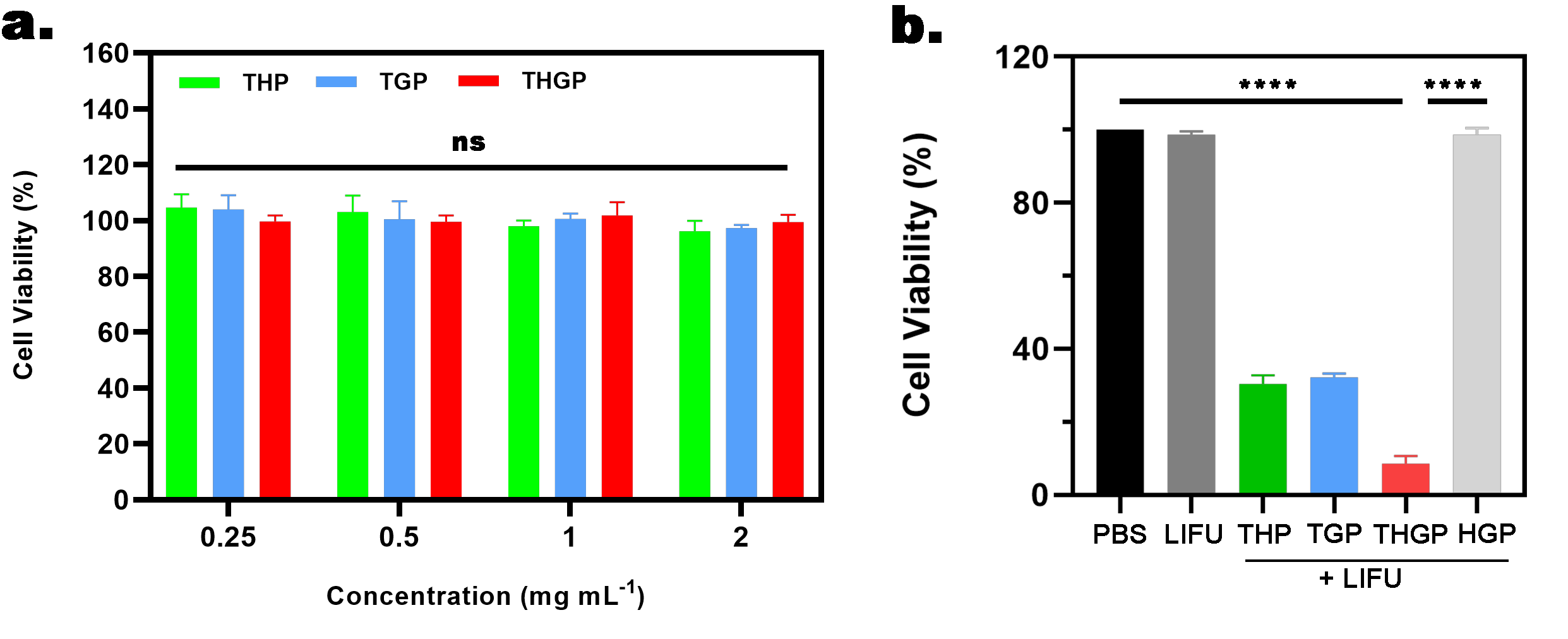


**Fig S8.** (a) Relative cell viability of TGP, THP and THGP groups with different concentrations. (b) Relative cell viability of different treatment groups.


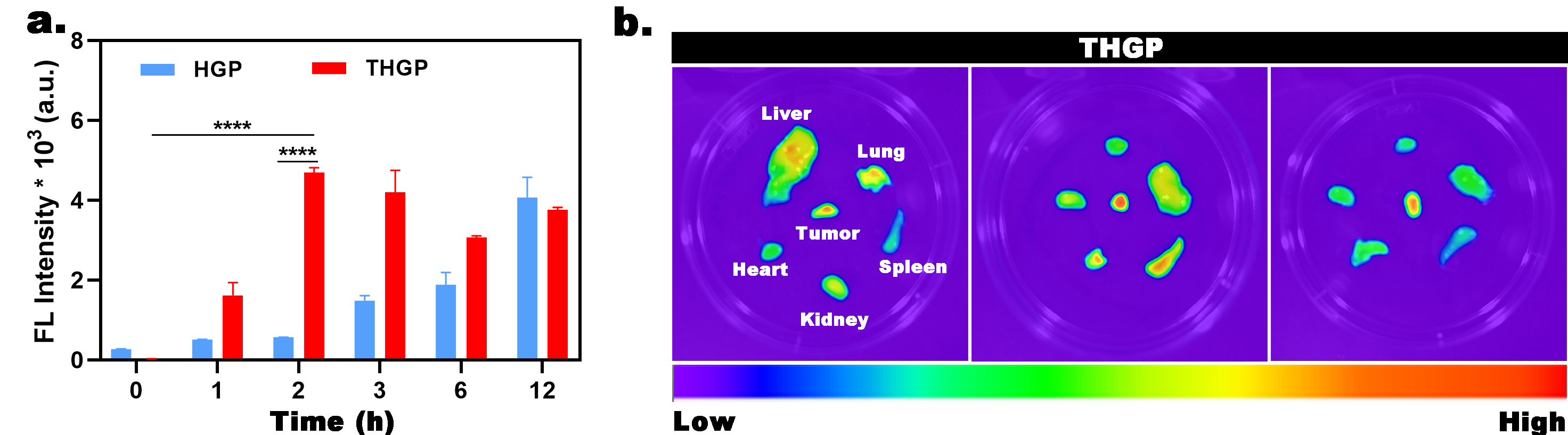


**Fig S9.** (a) *In vivo* metabolic FL intensity after intravenous injection with THGP and HGP for various time intervals. (b) FL imaging of excised major organs and tumors.


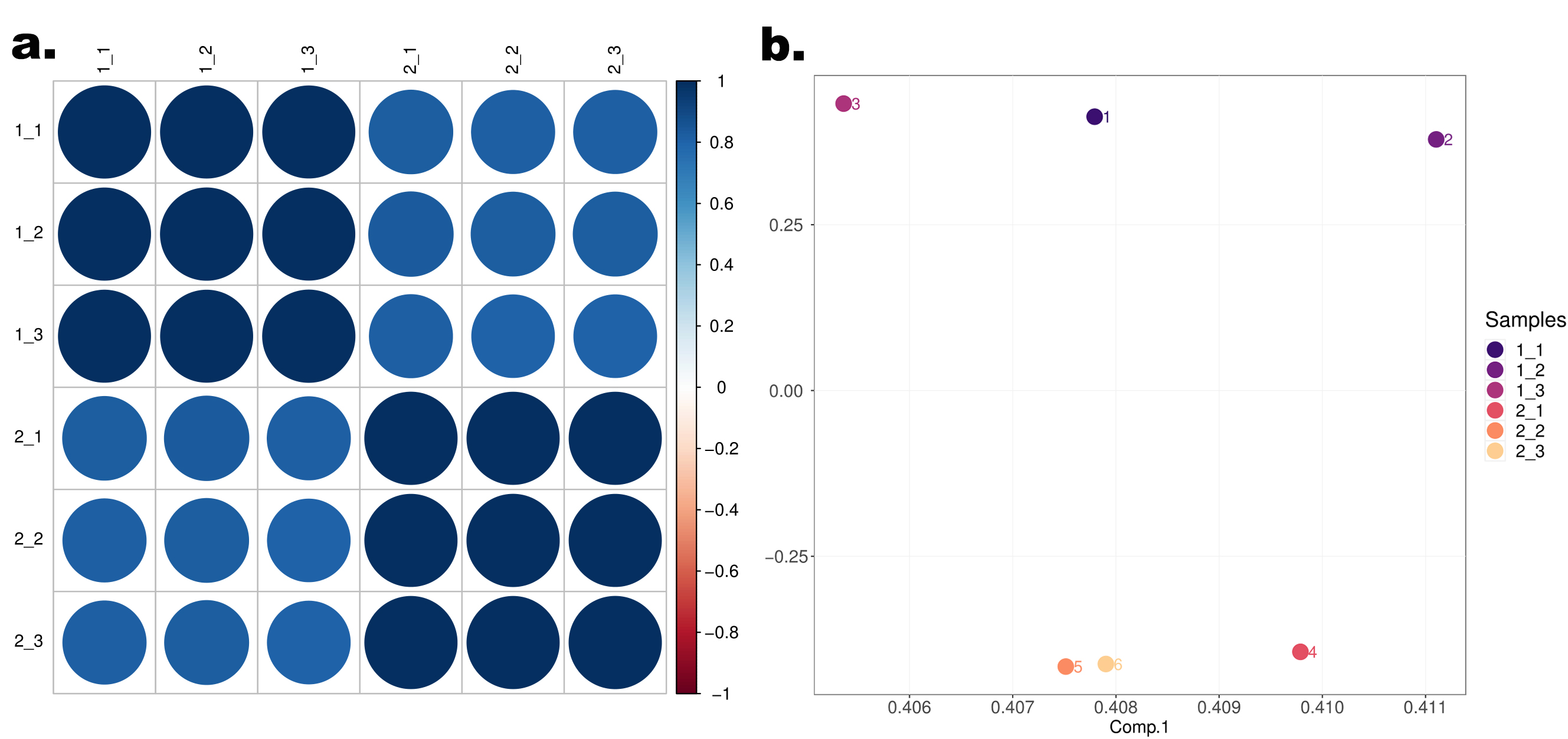


**Fig S10.** (a) Pearson correlation analysis. (b) PCA dimensionality reduction analysis.


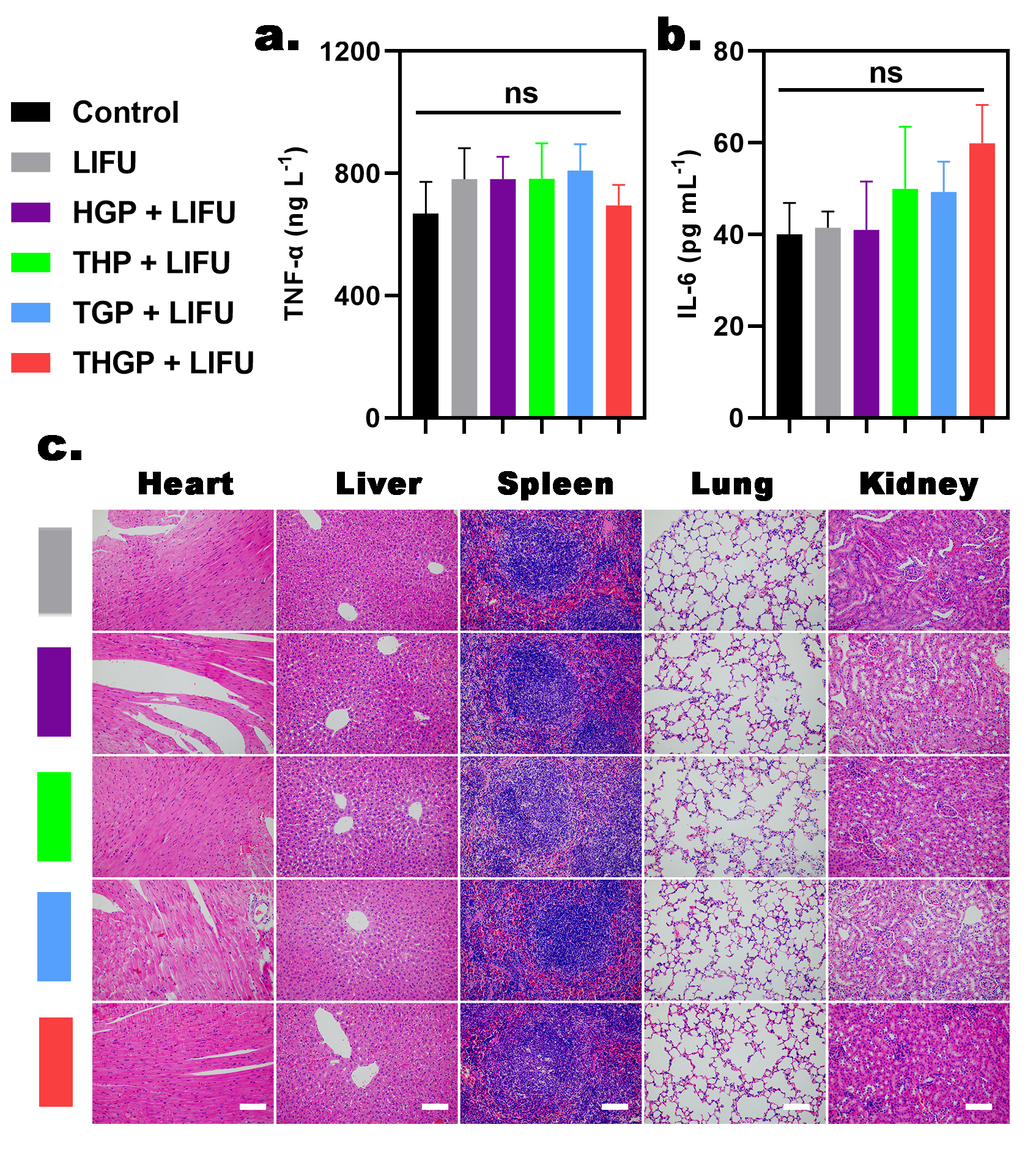


**Fig S11.** (a) and (b) Elisa of the levels of inflammatory cytokines of MDA-MB-231 tumor-bearing mice 16 days after administration of various treatments, including: TNF-α and IL-6. (c) H&E staining of the major organs 16 d after different treatments were administered.
